# Cell-type-specific epigenomic variations associated with *BRCA1* mutation in pre-cancer human breast tissues

**DOI:** 10.1093/nargab/lqac006

**Published:** 2022-02-02

**Authors:** Yuan-Pang Hsieh, Lynette B Naler, Sai Ma, Chang Lu

**Affiliations:** Department of Chemical Engineering, Virginia Tech, Blacksburg, VA 24061, USA; Department of Chemical Engineering, Virginia Tech, Blacksburg, VA 24061, USA; Department of Biomedical Engineering and Mechanics, Virginia Tech, Blacksburg, VA 24061, USA; Department of Chemical Engineering, Virginia Tech, Blacksburg, VA 24061, USA; Wake Forest Baptist Medical Center Comprehensive Cancer Center, Winston Salem, NC 27157, USA

## Abstract

*BRCA1* germline mutation carriers are predisposed to breast cancers. Epigenomic regulations have been known to strongly interact with genetic variations and potentially mediate biochemical cascades involved in tumorigenesis. Due to the cell-type specificity of epigenomic features, profiling of individual cell types is critical for understanding the molecular events in various cellular compartments within complex breast tissue. Here, we produced cell-type-specific profiles of genome-wide histone modifications including H3K27ac and H3K4me3 in basal, luminal progenitor, mature luminal and stromal cells extracted from a small pilot cohort of pre-cancer *BRCA1* mutation carriers (*BRCA1^mut/+^*) and non-carriers (*BRCA1^+/+^*), using a low-input ChIP-seq technology that we developed. We discovered that basal and stromal cells present the most extensive epigenomic differences between mutation carriers (*BRCA1^mut/+^*) and non-carriers (*BRCA1^+/+^*), while luminal progenitor and mature luminal cells are relatively unchanged with the mutation. Furthermore, the epigenomic changes in basal cells due to *BRCA1* mutation appear to facilitate their transformation into luminal progenitor cells. Taken together, epigenomic regulation plays an important role in the case of *BRCA1* mutation for shaping the molecular landscape that facilitates tumorigenesis.

## INTRODUCTION

Mutations on tumor suppressor gene *BRCA1* have been strongly linked to increased risks to breast, ovarian and other cancers (1). However, how these genetic alterations trigger the molecular cascades that ultimately lead to the pathology of tumorigenesis remains unclear. Breast tissue contains both epithelial and stromal compartments and the former can be further divided into basal (BCs), luminal progenitors (LPs), and mature luminal (MLs) cells based on their surface markers that are indicative of their developmental lineage and/or location in the two epithelial layers of the mammary duct (2). These various cell types present characteristic gene expression patterns and epigenomic landscapes (3–6). Breast tumors involving *BRCA1* germline mutation are predominantly basal-like/triple-negative (7–9). Recent results have suggested that *BRCA1*-associated basal-like breast cancers originate from luminal progenitor cells instead of basal stem cells (10,11). Thus, it is critical to understand how various cell types within breast tissue are affected by *BRCA1* mutation and how such dynamics in the cellular identity potentially contribute to tumorigenesis.

Epigenomic landscape plays a significant role in defining the cell state and mediating genetic factors into molecular cascades that are eventually involved in disease development. DNA sequence variation is known to impact epigenetic landscape, chromatin structures and molecular phenotypes via influencing the cis-regulatory elements such as promoters and enhancers (12–14). The changes in the epigenetic landscape may in turn alter gene expression and cellular phenotypes to promote cancer development. While traditional triple-negative breast cancer has been associated with increases in super-enhancers (15,16), *BRCA1* mutation has been recently shown to significantly attenuate epigenomic functional elements such as enhancers in our study using pre-cancerous breast tissue homogenates (17). However, due to predominant basal-like characteristics of *BRCA1*-associated tumors, cell-type-specific profiling of tissue samples is needed to decipher how each cell type within breast tissue is affected by the mutation and contributes to tumorigenesis.

In this study, we profile two important histone marks H3K4me3 and H3K27ac in a cell-type-specific manner in all four major cell types from pre-cancerous human breast tissue samples using a low-input ChIP-seq technology that we developed (MOWChIP-seq (18,19)). It is important to note that we define pre-cancerous breast tissue as healthy tissue from a breast with no history of cancerous growths. It does not intend to imply that this is tissue from a breast that will assuredly develop cancer. We compare the data on *BRCA1* mutation carriers (*BRCA1^mut/+^*) and non-carriers (*BRCA1^+/+^*) and extract epigenomic features that separate the two groups. Such comparison reveals that the extent of epigenomic changes varies among the four cell types. Despite a limited sample size, we find striking changes that warrant additional study and correlate our results with current literature. These epigenomic alterations potentially change the cell state and lay the groundwork for future tumorigenesis.

## MATERIALS AND METHODS

### Breast tissues

The study was approved by the Institutional Review Board at the University of Texas Health Science Center at San Antonio. Informed consent was obtained from all participants. Breast tissues were obtained from adult female cancer-free *BRCA1* mutation carriers (MUT) or non-carriers (NC) who underwent cosmetic reduction of mammoplasty, diagnostic biopsies or mastectomy. Genetic testing of *BRCA1* mutation was conducted by the hospital (20). The de-identified tissue samples were provided to the authors by Dr. Rong Li and Dr. Xiaowen Zhang of UTHSCSA. Analysis of the de-identified patient materials was approved by the Institutional Review Board of Virginia Tech. Using previously published protocols (21), fresh unfixed breast tissue was processed to generate single-cell suspension and the single cells were sorted into four fractions using FACS: EpCAM^_^CD49f^_^ stromal cells (SCs), EpCAMlowCD49fhigh basal cells (BCs), EpCAMhigh CD49f + luminal progenitor cells (LPs) and EpCAMhigh CD49f^_^ mature luminal cells (MLs).

### Chromatin shearing

The sonication process to generate chromatin fragments is similar to what we described in previous publications (18,19). A sorted cell sample of a specific type (containing 100K to 3 million cells, depending on the cell type and sample) was centrifuged at 1600 *g* for 5 min at room temperature and washed twice with 1 ml PBS (4°C). Cells were resuspended in 1 ml of 1% freshly prepared formaldehyde in PBS and incubated at room temperature on a shaker for 5 min. Crosslinking was quenched by adding 0.05 ml of 2.5 M glycine and shaking for 5 min at room temperature. The crosslinked cells were centrifuged at 1600 *g* for 5 min and washed twice with 1 ml PBS (4°C). The pelleted cells were resuspended in 130 μl of the sonication buffer (Covaris, 10 mM Tris-HCl, pH 8.0, 1 mM EDTA, 0.1% SDS and 1× protease inhibitor cocktail [PIC]) and sonicated with 105 W peak incident power, 5% duty factor, and 200 cycles per burst for 16 min using a Covaris S220 sonicator (Covaris). The sonicated chromatin samples were shipped to Virginia Tech for MOWChIP-seq assay. The sonicated sample was centrifuged at 16 100 *g* for 10 min at 4°C. The sheared chromatin in the supernatant was transferred to a pre-autoclaved 1.5 ml microcentrifuge tube (VWR). A fraction of the sonicated chromatin sample was mixed with IP buffer (20 Mm Tris-HCl, pH 8.0, 140 mM NaCl, 1 mM EDTA, 0.5 mM EGTA, 0.1% (w/v) sodium deoxycholate, 0.1% SDS, 1% (v/v) Triton X-100, with 1% freshly added PMSF and PIC) to generate a MOWChIP sample containing chromatin from 50 000 cells with a total volume of 50 μl.

### MOWChIP-seq

We conducted MOWChIP-seq of the sonicated chromatin samples with 50 000 cells per assay for H3K27ac profiling and 10 000 cells per assay for H3K4me3 profiling, using protocols and microfluidic devices described in our previous publications (18,19). We used anti-H3K27ac antibody (abcam, cat: ab4729, lot: GR323132-1) and anti-H3K4me3 antibody (Millipore, cat: 07–473, lot: 2930138) in these experiments.

### Data quality control

ChIP-seq data sets that had fewer than 10 000 called peaks in a given technical replicate were discarded. After quality control, the technical replicates of the same cell sample were combined for the data analysis. As a result, we obtained three biological replicates for MUT H3K27ac samples, four biological replicates for NC H3K27ac samples, two biological replicates for MUT H3K4me3 samples and one biological replicate for the NC H3K4me3 sample. The fraction of reads in peaks (FrIP) was calculated using the number of mapped reads within peak regions divided by total mapped reads. Normalized-strand correlation (NSC) and relative-strand correlation (RSC) were calculated using phantompeakqualtools v1.2.2 (22,23).

### Data processing

Unless otherwise mentioned, all data analysis was performed with Bash scripts or with R v3.6.1 (The R Foundation) scripts in RStudio. Sequencing reads were trimmed using default settings by Trim Galore! v0.4.1 (Babraham Institute). Trimmed reads were aligned to the hg19 genome with Bowtie v1.1.2 (24). Peaks were called using MACS2 v2.1.1.20160309 (*q* < 0.05) (25). Blacklisted regions in hg19 as defined by ENCODE were removed to improve data quality (26). Mapped reads from ChIP and input samples were extended by 100 bp on either side (250 bp total) and a normalized signal was calculated.

$$\begin{eqnarray*} && {\rm Normalized}\ {\rm Signal }\nonumber \\ && \quad = \left( {\frac{{{\rm ChIP}\ {\rm Signal}}}{{{\rm No.}\ {\rm of}\ {\rm ChIP}\ {\rm Reads}}} - \frac{{{\rm Input}\ {\rm Signal}}}{{{\rm No.}\ {\rm of}\ {\rm Input}\ {\rm Reads}}}} \right) \times {10^6} \end{eqnarray*}$$

For Pearson’s correlation, the signal was calculated around the promoter region (TSS ± 2 kb) and plotted with the corr and levelplot functions. For visualization in IGV v2.4.10 (Broad Institute), the signal was calculated in 100 bp windows over the entire genome and output as a bigWig file.

### Differential analysis

To determine peak regions with differential signal, the Bioconductor package DiffBind v2.12.0 was used (27,28). A ‘majority-rules’ consensus peak set was generated for each experimental group and combined to make a master set for analysis. Peaks were considered to be valid if they were present in the majority of biological replicates. Counts were generated using default conditions and compared using the DESeq2 option. Normalized signal counts were extracted and plotted in heatmaps and boxplots using ggplot2 v3.3.1 (29). Gene ontology analysis was performed using the web-based tool GREAT v4.0.4 (30) with default settings for hg19. For the SC analysis, the top 6000 regions (by smallest FDR value) were used.

### Enhancers analysis

To call enhancers, we considered H3K27ac^high^ regions that did not intersect with promoter regions to be enhancer regions. First, consensus H3K27ac peak sets were generated for NC and MUT samples for each cell type after determining the set of peak regions present in NC and/or MUT samples. Peak widths were expanded to be 1000 bp long (summit ± 500 bp). Promoters were defined as TSS ± 500 bp. Any H3K27ac 1 kb regions that intersected with a promoter region was removed and the remaining regions were designated as enhancers. Motif analysis was performed to determine enriched transcription factor binding motifs among the enhancer regions with HOMER v4.10.3 (31) (with options –size 1000 –mask –p 16 –nomotif). Functional classification of transcription factors was performed using Panther v15.0.0 (32). Enhancers were mapped to genomic regions with ChIPSeeker v1.20.0 (33). Enhancers were considered associated with ER-negative SNPs (obtained from NHGRI-EBI GWAS Catalog (34)) if the SNP was within 150 kb up- or downstream.

## RESULTS

**Figure 1.**
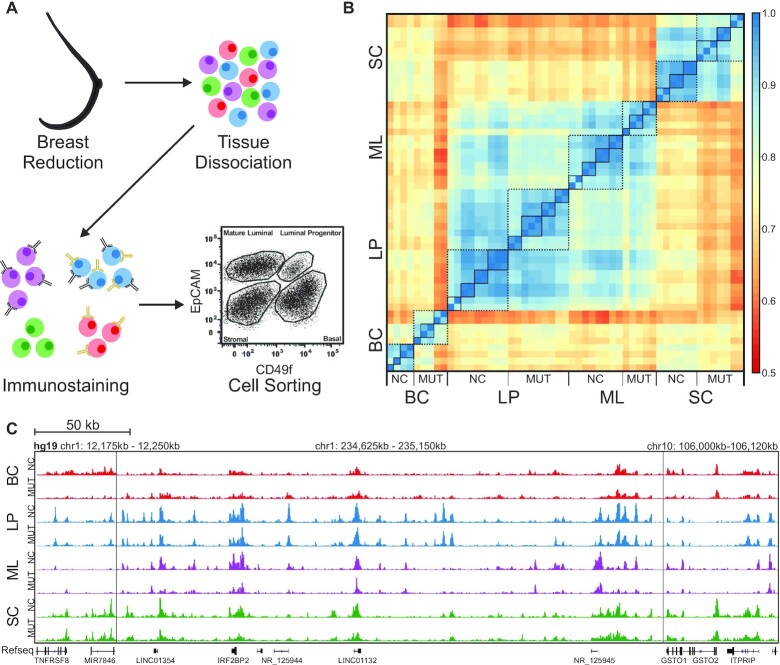
Cell-type-specific ChIP-seq data on human breast samples from *BRCA1* mutation carriers (MUTs) and non-carriers (NCs). (**A**) Breast tissue samples were separated into the basal cells (BCs), luminal progenitor cells (LPs), mature luminal cells (MLs) and stromal cells (SCs) by FACS. Flow plot is a schematic of typical NC flow cytometry data. (**B**) Pearson correlations among H3K27ac ChIP-seq data sets of various cell types from NCs and MUTs around promoter regions (TSS ± 2 kb). Each solid-line frame circles the technical replicates on one cell sample and each broken-line frame circles the data on a specific cell type. (**C**) Representative tracks of normalized H3K27ac signal for each of the cell types from NCs and MUTs. Three regions in the genome are presented and separated by vertical lines.

Breast tissues from *BRCA1* mutation carriers (MUTs, *n* = 3 for H3K27ac, 2 for H3K4me3) and non-carriers (NCs, *n* = 3–4 for H3K27ac, 1 for H3K4me3) were collected during breast reduction or mastectomy surgery (Supplementary Table S1), dissociated, and sorted into the basal, luminal progenitor, mature luminal and stromal cell (SC) types (Figure 1A and Supplementary Table S2) (35). Due to the nature of the tissue collection requirement, tissue availability was a concern and was responsible for our small sample sizes. Regardless, the results we present here can still be important as a means of instigating further research into an unsettled topic. We profiled H3K4me3 and H3K27ac using MOWChIP-seq with at least two technical replicates for each cell sample (Supplementary Tables S3 and S4). We selected H3K4me3 as it is an activating mark that is associated with transcriptional start sites (TSSs) of genes (36,37), and H3K27ac as it labels active enhancers (38). Both marks are positively correlated with increases in gene expression. All samples had a fraction of reads in peaks (FrIP), normalized-strand correlation (NSC) and relative-strand correlation (RSC) that fell within ENCODE guidelines (23). Average NRF values for H3K27ac samples and H3K4me3 samples were 0.87 and 0.83, respectively, and are ENCODE compliant. Each replicate was normalized to account for differences in sequencing depth. Similarly, input data were also normalized to sequencing depth before subtraction from sample data for input normalization. Our ChIP-seq datasets are highly correlated between technical replicates with an average Pearson correlation coefficient r of 0.962 for H3K4me3 and 0.950 for H3K27ac. We generally define the correlation to be considered high when *r* > 0.95, good when *r* > 0.9, fair when *r* > 0.75, low when *r* < 0.75 and poor when *r* < 0.6. We also observed very high genome-wide correlations among biological replicates in a group (MUTs or NCs), with an average *r* of 0.960 for H3K4me3 and 0.918 for H3K27ac (Figure 1B). Generally, H3K4me3 is not a strong differentiating mark for separating MUTs and NCs. The correlation r between NCs and MUTs H3K4me3 data is high for all cell types (0.962 for BCs, 0.960 for LPs, 0.962 for MLs and 0.960 for SCs) (Supplementary Figure S1). In contrast, when genome-wide H3K27ac is examined, many more differential peaks are observed between MUTs and NCs and among various cell types (Figure 1C). BCs and SCs show the largest differences between MUTs and NCs (with an average *r* of 0.739 and 0.877, respectively). In comparison, LPs and MLs have similar H3K27ac profiles between MUT and NC (with an average *r* of 0.914 and 0.888, respectively).

In terms of differences among various cell types, BCs, LPs, and MLs have very similar H3K4me3 profiles (average *r* of 0.926 among MUTs and 0.946 among NCs) while SCs show slightly more differences from LPs and MLs (average *r* of 0.881 and 0.915 between SCs and LPs, 0.875 and 0.919 between SCs and MLs in MUT and NC, respectively). With H3K27ac data, LPs and MLs correlate with each other fairly well (average *r* = 0.832 and 0.872 in MUTs and NCs, respectively) while the other pairs generally have low correlation (with average *r* in the range of 0.668–0.794).

**Figure 2.**
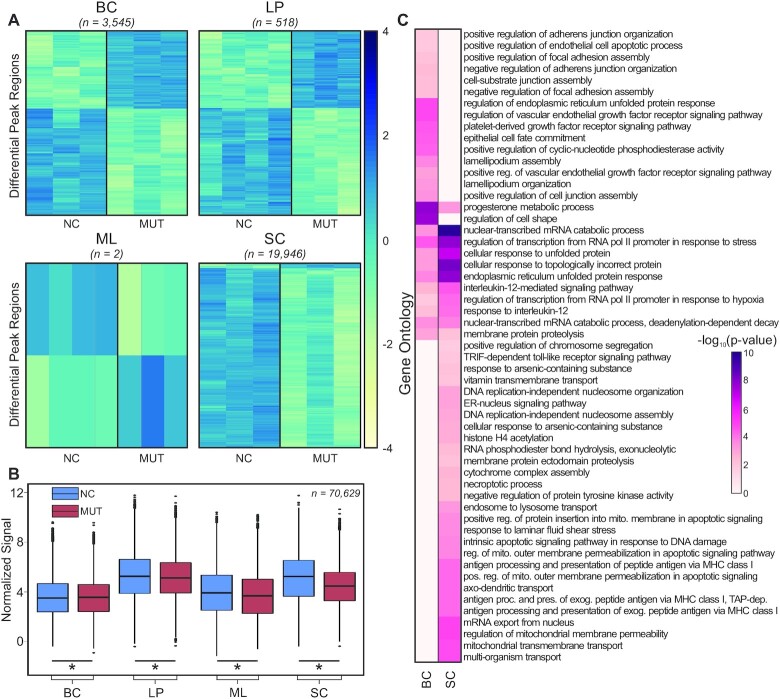
Differential H3K27ac peak regions between NCs and MUTs. (**A**) Heatmaps of differentially modified H3K27ac peak regions found to be significant (fold-change ≥ 2, FDR < 0.05) between NCs and MUTs. (**B**) Boxplots of normalized H3K27ac signal at all peak regions (*n* = 70 629 peaks). Asterisks (*) denote significant difference (*P* < 0.05, paired Student’s *t*-test). (**C**) Gene ontology enrichment analysis using GREAT of significant differentially modified peak regions between NCs and MUTs.

We carefully examined differentially modified H3K27ac peak regions (fold-change ≥ 2, FDR < 0.05) between MUTs and NCs (Figure 2A). We found very few differential regions in LPs and MLs (518 and 2, respectively). However, there were a substantial number of differential peaks present in BCs (3545) and a large number of different peaks in SCs (19 946). BCs had a mix of regions that showed either higher or lower H3K27ac signal in MUTs than in NCs (1497 and 2048, respectively), while the vast majority of differential regions in SCs (19 367 out of 19 946) had lower H3K27ac signal in MUT samples. We then compared the normalized H3K27ac signal at all peak regions (Figure 2B). The median values were similar between NC and MUT patients in all epithelial cell types (with MUT values within ± 5% of NC ones), while there was a marked decrease in H3K27ac median signal in MUT SCs (by 13.5% compared to NC SCs). In these comparisons, all differences were found to be statistically significant (*P* < 0.05, paired Student’s *t*-test).

The differentially modified H3K27ac regions were then mapped to their nearest genes (Supplementary Tables S5–S7). Due to the complex nature of activation by epigenetic modification, the differentially modified regions were not separated into up- and down-regulated regions for the gene ontology analysis. Thus, the analysis focuses on processes and pathways that have perturbed epigenetic modification due to *BRCA1* mutation. These differential regions were associated with 783, 3972 and 12 160 genes in LPs, BCs and SCs, respectively. We also conducted gene ontology enrichment analysis using these regions for each of the cell types (Supplementary Table S8). The analysis on LPs and on MLs did not bring out any GO terms. Thus, our focus was on BCs and SCs which had the largest numbers of differential H3K27ac regions between NCs and MUTs (Figure 2C). A number of BRCA1-associated processes, including progesterone metabolism (39–42), RNA polymerase II transcription (43–45) and unfolded protein response (UPR) (46), were enriched in both BCs and SCs. BRCA1 has been shown to inhibit progesterone signaling (42) and reduction in BRCA1 level has been shown to increase expression of GRP78, a key UPR regulating gene (46). Moreover, BRCA1 is part of the RNA polymerase II holoenzyme (43). Ontologies related to apoptosis (47,48), antigen processing (49–51) and DNA damage response (52,53) are only significant in SCs. BRCA1 has extensive association with apoptosis, including those due to endoplasmic reticulum stress that is related to UPR (54). For example, BRCA1 binding at the endoplasmic reticulum leads to a release of calcium that causes apoptosis. Furthermore, a reduction in BRCA1 level has been shown to increase activation of CD8^+^ tumor-infiltrating lymphocytes. There are also several ontologies associated with DNA damage response significant in SCs. For instance, DNA replication-independent nucleosome assembly and organization can only occur with histone variant 3.3, which is part of the DNA repair pathway (55,56). In addition, histone H4 acetylation also opens up the chromatin for easier access to damaged regions (57). In contrast, we largely see ontologies associated with cell motility and adhesion in BCs. *BRCA1* mutations have been shown to increase cell motility in cancer cells (58–60). However, epithelial cell fate commitment is also present only in BCs. Cells within the BC compartment have been previously shown to have the potential to differentiate into LPs (61).

**Figure 3.**
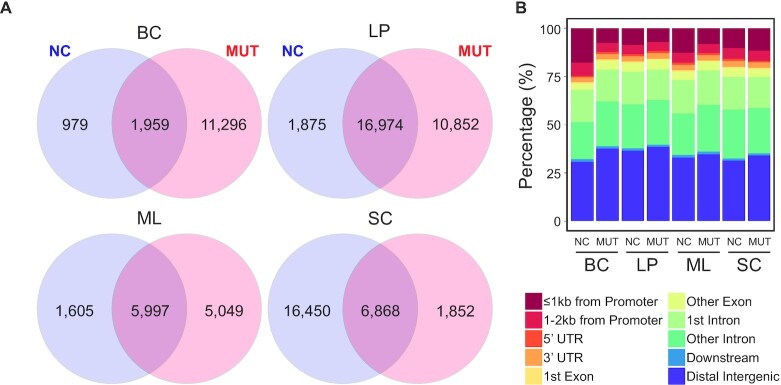
Enhancers predicted in various cell types from NCs and MUTs. (**A**) Venn diagrams of overlap between NC and MUT enhancers. (**B**) Genomic locations of the enhancers in various cell types.

Next, we predicted enhancers present in each of the cell types for both NC and MUT samples (Figure 3A). Enhancers were determined by finding H3K27ac^high^ regions that did not intersect with areas nearby transcription start sites (±500 bp from TSS). For the purposes of this analysis, we have defined the region within 500 bp of the TSS as the promoter region. Using the NCs as the reference, MUT enhancers cover 67%, 90%, 79% and 29% of the NC enhancers in BCs, LPs, MLs and SCs, respectively. Furthermore, while MUT BCs and LPs had a similar number of unique enhancers, 85% of MUT BC enhancers were unique compared to 39% of MUT LP enhancers, supporting the notion that *BRCA1* mutation affects BCs more than LPs. The enhancers were then mapped to genomic regions (Figure 3B and Supplementary Table S9). The most exaggerated differences due to *BRCA1* mutation were seen in BCs, including a 6.9% increase in the distal intergenic fraction and 12.3% decrease in the promoter vicinity fraction (i.e. <2 kb from the edge of the promoter regions). In addition, we also found that super enhancers are significantly attenuated in all cell types except for BCs, in congruence with our previous homogenate data (Supplementary Figure S2). It is clear that *BRCA1* mutation plays a different role in enhancer activity that is unique to each cell type.

**Figure 4.**
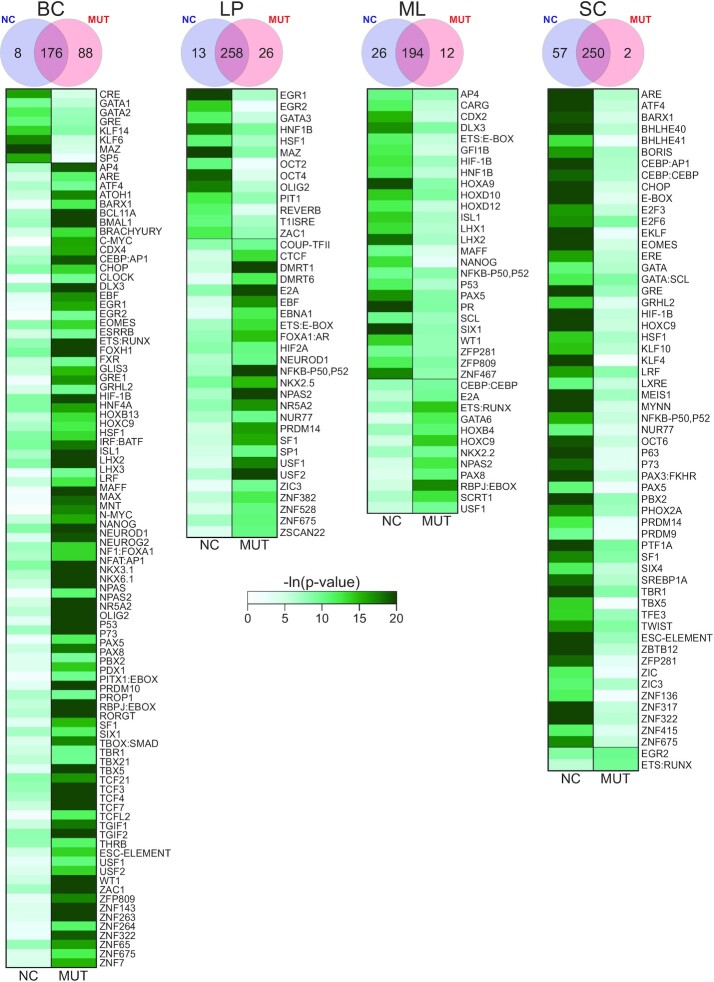
Heatmaps of motifs found to be significantly enriched in either NC or MUT. Color represents the level of significance. Venn diagrams of overlap between motifs enriched in NCs or MUTs are also presented.

Enhancer regions were then scanned to determine the transcription factor (TF) binding motifs significantly enriched in each cell population (Figure 4 and Supplementary Table S10). BCs had substantially more differentially enriched TF motifs than any other cell type, likely due to the smaller overlap between NC and MUT enhancers for BCs. We defined differentially enriched TFs as those that are uniquely enriched in one condition, but not the other. In MUT samples, BCs primarily have many additional TFs, while SCs mainly lacked TFs. In all cases, over 50% of differentially enriched TFs were found to have no known link to *BRCA1* mutation. However, some TFs were found to be differentially enriched between MUT and NC in multiple cell types with known association with *BRCA1* mutation. These include *PAX5* (62) (BCs, MLs and SCs), *CHOP* (46) (BCs and SCs), *EGR1* (63,64) (BCs and LPs) and *P73* (65) (BCs and SCs). Some TFs found were not associated with *BRCA1* yet were associated with breast cancer. These include *EGR2* (66,67) (BCs, LPs and SCs), *HOXC9* (68,69) (BCs, MLs and SCs), *HSF1* (70–72) (BCs, LPs and SCs), *NPAS2* (73) (BCs, LPs and MLs) and *USF1* (74) (BCs, LPs and MLs). We then used PANTHER to classify the combined differential TFs from each cell type comparison into pathways and found that pathways such as Gonadotropin-releasing hormone (GnRH) receptor pathway, Wnt signaling pathway, apoptosis pathway and p53 pathways were present in all four cell types (Supplementary Table S11). *BRCA1* has a key role in the Wnt signaling pathway (75,76), regulates apoptotic responses (47,48) and has been shown to interact with *P53* (77–80). As for the GnRH pathway, while there is not a direct link to BRCA1, GnRH agonists have been shown to be effective in the treatment of breast cancer (81).

**Figure 5.**
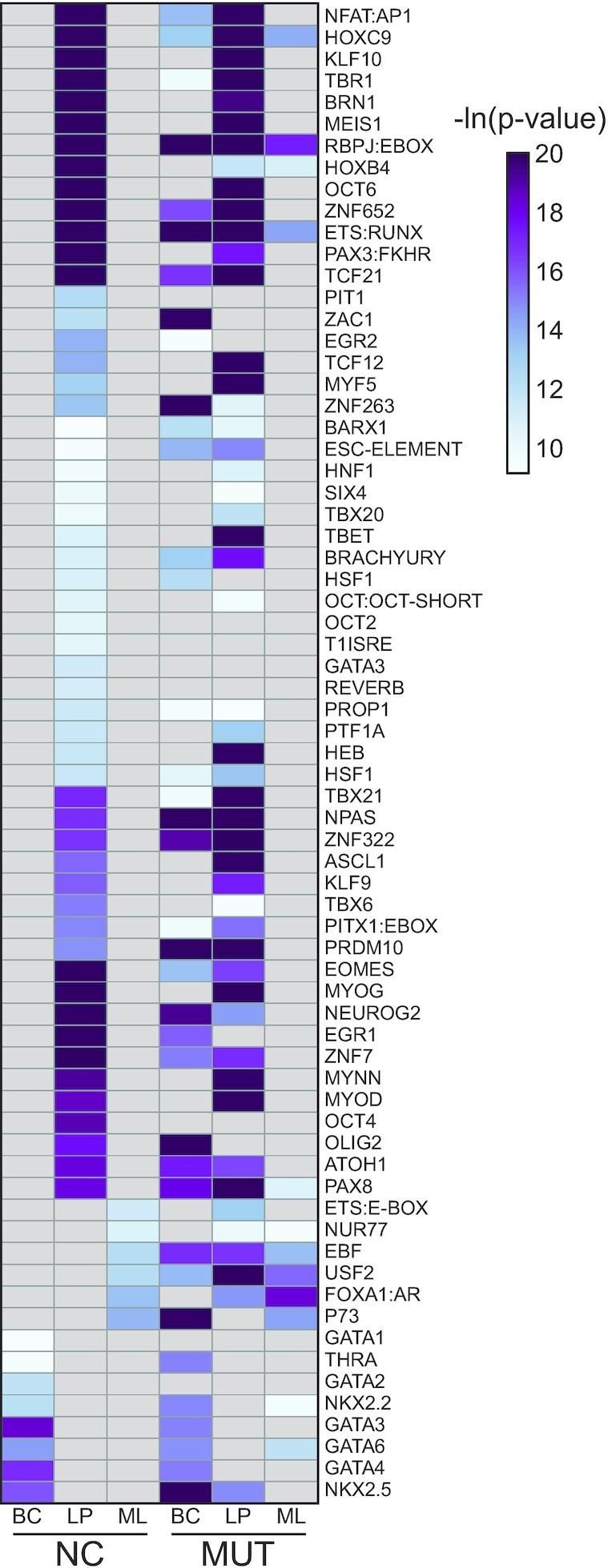
Heatmap of *P*-values for cell-type-specific motifs in NC and MUT. *P*-values of motifs found significantly enriched (*P*-value < 0.0001) in only one of three NC cell types (BCs, LPs or MLs) from NCs and their enrichment in MUTs. Color represents the level of significance. All *P*-values that are not significant are colored gray.

Overall, we see the most significant epigenomic changes in BCs and SCs due to *BRCA1* mutation among the four cell types from these pre-cancer breast tissue samples, while fewer variations were seen in LPs. This is unexpected as LPs have been implicated as the driver in the onset of *BRCA1* mutation associated breast cancer (82). Thus, we examine the possibility of basal cells differentiating into luminal cells, as proposed in previous literature (61). First, we examined the cell-type specific genes identified for the three epithelial cell types (BCs, LPs and MLs) in the literature (4). The expression of these genes largely defines the identity of specific cell types. By comparing NC and MUT BCs, we found that 6% (41/712) of the BC-specific genes experiences significant changes in H3K27ac state due to the mutation, compared to 1% (4/305) for LPs and 2% (11/444) for MLs (Supplementary Table S12). Of these differentially marked basal genes, 95% had lower H3K27ac signal in MUT, suggesting that there is primarily a loss of basal gene expression in MUT basal cells. In the same fashion, we also examined cell-type-specific TFs in the three cell types and how they vary due to the mutation. Enriched TFs in each cell population were predicted based on motif analysis of enhancers profiled using H3K27ac data (83). By examining NC samples, we extract 8, 55 and 6 cell-type-specific TFs (*P*-value < 0.0001) for BCs, LPs, and MLs, respectively (Figure 5 and Supplementary Table S13). These cell-type-specific TFs are TFs that are uniquely enriched in one NC cell type but not in the others. In comparison, MUT BCs, LPs and MLs preserved 6, 44 and 5 of these TFs, respectively. Interestingly, MUT BCs also enriched 28 of the LP-specific TFs and 3 of the ML-specific TFs, compared to MUT LP enriching 1 BC-specific TFs and 5 ML-specific TFs; and MUT ML enriching 2 BC-specific TFs and 5 LP-specific TFs. These results indicate that the BC state experiences more substantial change than LPs and MLs due to *BRCA1* mutation, consistent with the notion of BC differentiation into LPs.

**Figure 6.**
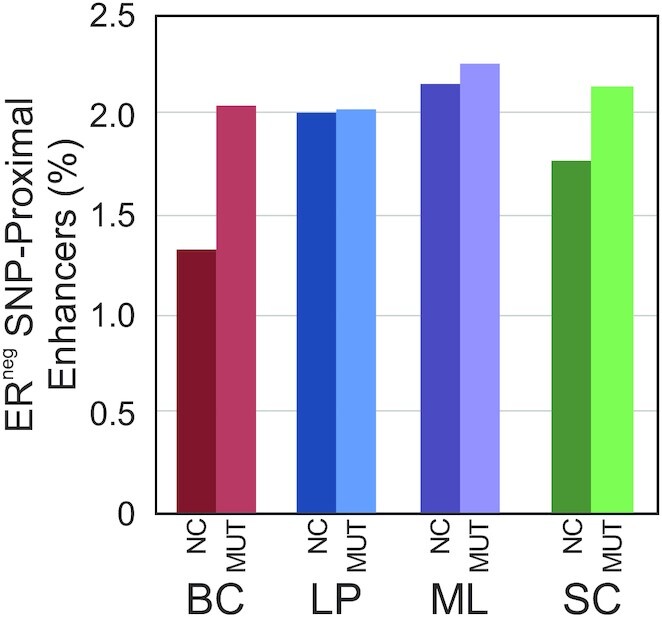
Percent of enhancers that are within ±150 kb of SNPs significantly associated with ER-negative breast cancers through GWAS for all four cell types.

To further validate the possibility of the basal compartment being a significant contributor in *BRCA1*-mutation associated breast cancer, we determined the number of enhancers that were in proximity to 123 SNPs involved in ER-negative breast cancer as discovered by GWAS (34) (Figure 6). ER-negative breast cancer is a term that effectively covers both basal-like and triple-negative breast cancers (84,85). Thus, we used the ER-negative SNP set due to its strong connection to BRCA1 breast cancer. In a cohort of 3797 *BRCA1* mutation carriers diagnosed with breast cancer, 78% had ER-negative breast cancers (86). Overall, we saw increases in the percent of enhancers that were proximal to ER-negative SNPs in each cell type due to *BRCA1* mutation, suggesting an overall increase in breast cancer risk. Furthermore, we see the largest increase (∼55%) between NC and MUT BCs followed by SCs (∼21%). This further supports that the *BRCA1* mutation leads to profound epigenetic changes in BCs and that these changes have the potential to increase breast cancer risk.

## DISCUSSION

The interactions between genomics and epigenomics are well-recognized events. Gene mutation may alter the epigenomic landscape in a significant way and such alternation may carry important implications on cancer development. Several lines of evidence support the feasibility of sorting out epigenomic differences between *BRCA1* mutation carriers and non-carriers using a cell-type specific approach. First, we found very high correlations among biological replicates in both H3K4me3 and H3K27ac within either MUT or NC group. When we compare across the two groups (MUT versus NC), H3K27ac is more differentiating than H3K4me3, which is consistent with previous findings by Ma *et al.* (83) and Roadmap Epigenomics *et al.* (87). Second, we compared our NC data with published results obtained by examining normal breast tissues pooled from multiple individuals (4). Each of the top 5 significantly enriched transcription factors in BCs, LPs and MLs were also significantly enriched in our respective NC cell populations. Basal-associated transcription factors (4) such as TP53, TP63, STAT3 and SOX9 were also enriched in NC BCs. Similarly, luminal-associated transcription factors (88–90) CEBPB, GATA3, ELF5 and FOXA1 were all significantly enriched in our NC LP and MLs. Third, we found that three members of the GATA family (GATA1, GATA2 and GATA3) were enriched in either NC BCs or LPs but not in MUT epithelial cells (Figures 4 and 5). This agrees with our earlier study conducted using breast tissue homogenates (17). GATA3 is known to be critically involved in the regulation of luminal cell differentiation (91,92). Finally, we also compared our cell-type-specific data with our published data on breast tissue homogenates obtained using a separate patient cohort and conventional ChIP-seq technology (17) (Supplementary Figure S3). The H3K27ac data taken using the breast tissue homogenate (mix) do not differentiate MUT and NC. The homogenate data show a similar degree of correlations with all individual cell types (Pearson correlation in the range of 0.741–0.890). This also underscores the importance for cell-type-specific profiling to pinpoint specific roles for each cell type. These comparisons suggest that although epigenomic differences exist among individual humans (93,94), careful cell-type-specific ChIP-seq profiling captures important genome-wide epigenomic differences due to *BRCA1* mutation. However, the small sample size limited our ability to adequately control confounding factors, such as age or environment. In addition, more subjects would improve the power of the analysis. Despite this, the consistency of our data with other sources support the substantive nature of our findings and strongly warrant further investigation.

Epigenetic profiles define cell identity by regulating cell-type-defining genes and transcription factors. The difference in the epigenomic landscape between *BRCA1* mutants and non-carriers may be important for explaining the high propensity of *BRCA1* mutation carriers for breast cancer. Our data on the sensitive mark H3K27ac are the most different in BCs and SCs when MUTs and NCs are compared. In comparison, very few changes were observed in LPs and MLs due to the mutation. Furthermore, our analysis of the cell-type-specific genes and TFs also reveal that MUT BCs resemble LPs. Such resemblance was in accordance with previous reports on the presence of LP-fated cells and bi-potent mammary stem cells in the basal compartment (61,95). Thus, we propose that the precancerous process within *BRCA1* mutation carriers may start with substantial epigenomic changes in basal cells among all epithelial cell types and these basal cells share similarity with luminal progenitor cells. These findings provide new insights into epigenomic factors involved in *BRCA1* cancer biology.

## DATA AVAILABILITY

The ChIP-seq datasets supporting the conclusions of this article are available in the Gene Expression Omnibus (GEO) repository with the accession number of GSE148995.

## Supplementary Material

lqac006_Supplemental_Files
