# Supplementary material for: Cell-type-specific epigenomic variations associated with BRCA1 mutation in pre-cancer human breast tissues
Source: NAR Genom Bioinform. 2022 Feb 2;4(1):lqac006. doi: 10.1093/nargab/lqac006 (PMC8808540; doi:10.1093/nargab/lqac006)
Supplement: lqac006_Supplemental_Files [file lqac006_supplemental_files.zip › Supplementary_Figures and Tables_12-12_final.pdf]

Supplementary Figures

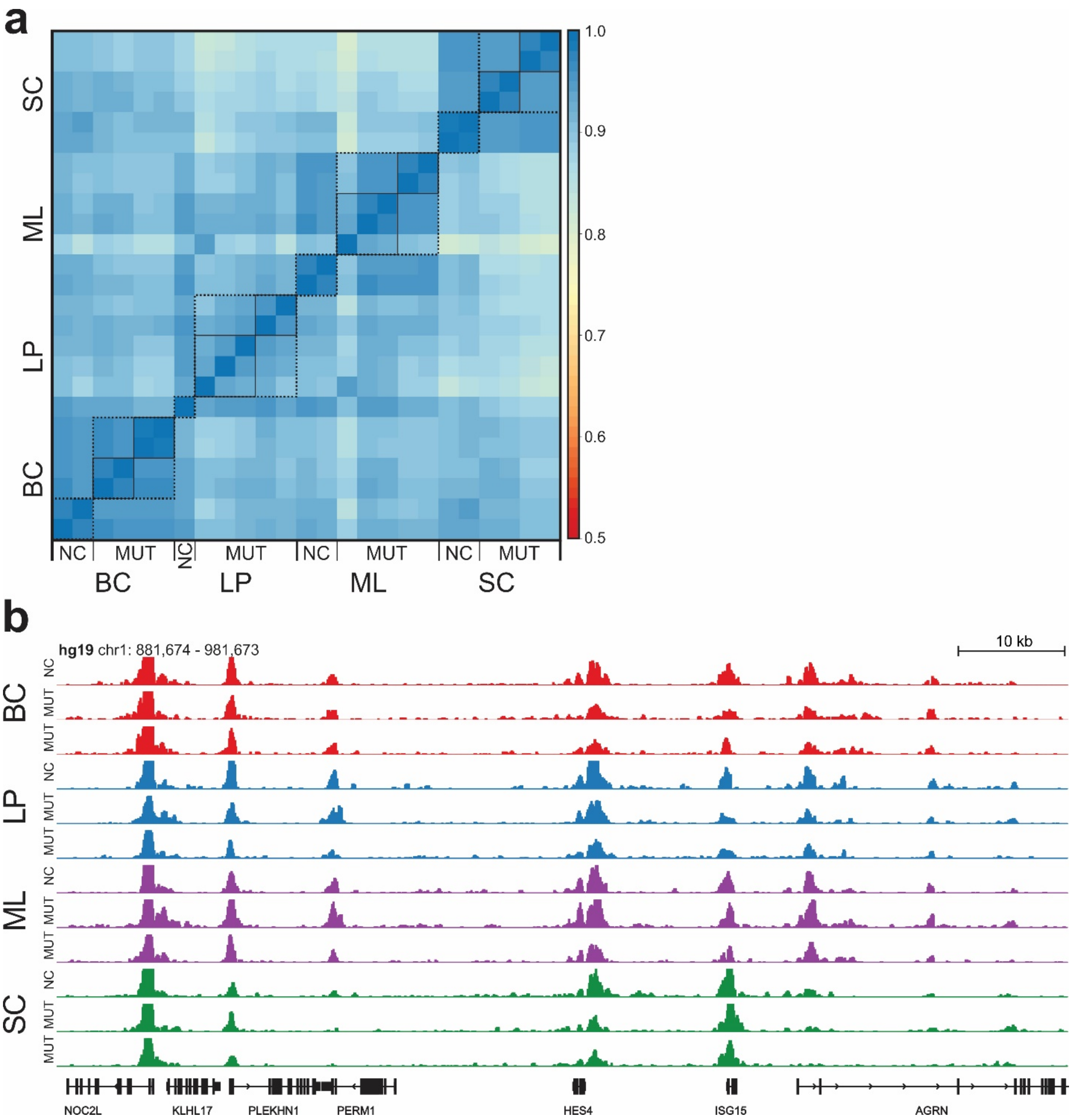

Figure S1 Cell-type-specific H3K4me3 ChIP-seq data on human breast samples from BRCA1 mutation carriers (MUTs) and non-carriers (NCs). (a) Pearson correlations among H3K4me3 ChIP-seq data sets of various cell types from NCs and MUTs around promoter regions (TSS +/- 2kb). Each solid-line frame circles the technical replicates on one cell sample and each broken-line frame circles the data on a specific cell type. (b) Representative tracks of normalized H3K4me3 signal for each of the cell types from NCs and MUTs.

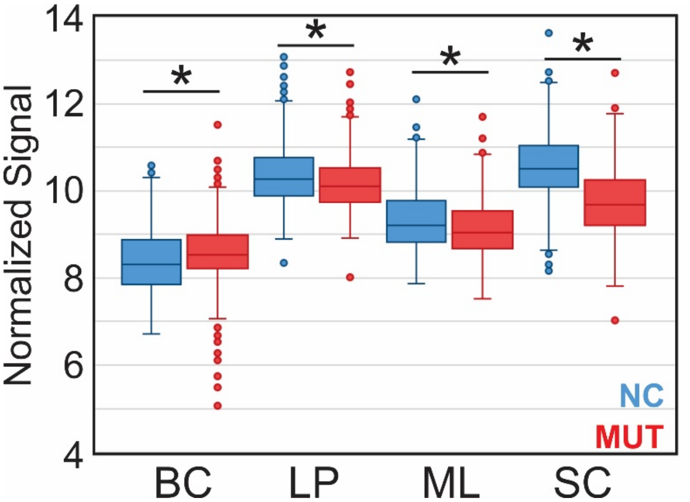

Figure S2 Normalized H3K27ac signal at super enhancers present in either the NC or MUT population for each cell type. Asterisk denotes significance ($p < 0.05$, paired Student's t-test).

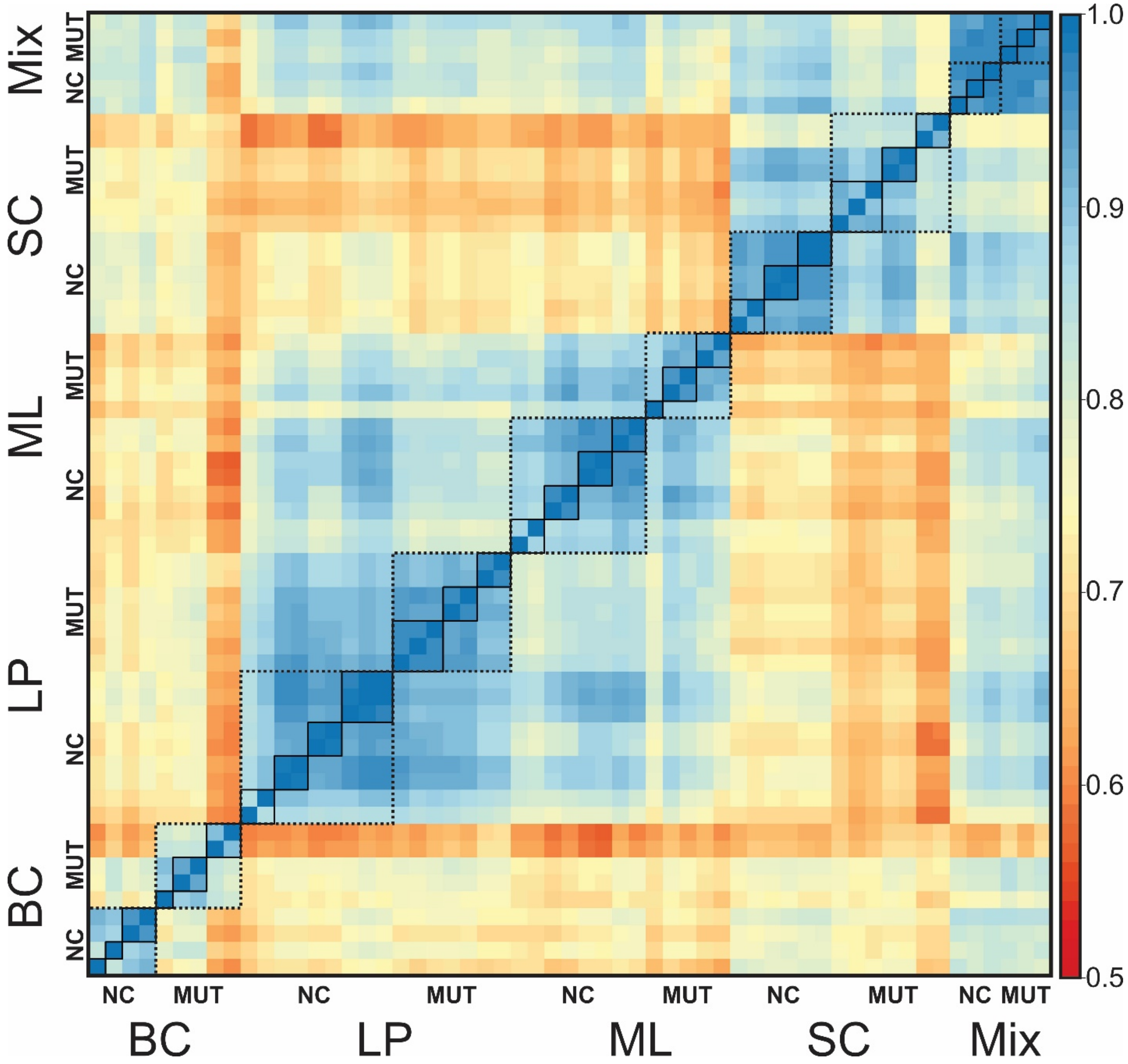

Figure S3 Genome-wide correlations among cell-type-specific and homogenate H3K27ac data. "Mix" refers to H3K27ac data (Ref. 15, GSE121229 from GEO) obtained using human breast tissue homogenates containing epithelium-enriched cell population.

Supplementary Tables

Table S1: Donor Characteristics

Donor	Age	Ethnicity	Menstrual Status	Mutation
NC1	45	Hispanic	Pre	N/A
NC2	54	White	Post	N/A
NC3	45	Hispanic	Pre	N/A
NC4	56	Hispanic	Post	N/A
NC5	34	Hispanic	Pre	N/A
MUT1	35	Hispanic	Pre	BRCA1 mutation (c.70_80del [p.Cys24Serfs*13], high cancer risk)<br>APC mutation (c.6790G>T [p.Gly2264Cys], uncertain clinical significance)
MUT2	35	Native American	Pre	BRCA1 mutation (c.5123C>A)<br>MSH6 mutation (c.719G>A, uncertain significance)
MUT3	27	Hispanic	Pre	BRCA1 mutation (deletion exon 23)

Table S2: Percentage of breast cell-type populations obtained by FACS

Donor	Population (%)			
	BC	LP	ML	SC
NC1	28.2	4.2	11.0	44.7
NC2	19.3	18.9	10.6	43.2
NC3	6.3	38.4	15.7	28.8
NC4	33.9	2.3	15.7	41.1
NC5	23.8	17.1	5.8	23.11
MUT1	31.6	17.6	21.0	20.4
MUT2	15.8	33.3	7.1	33.3
MUT3	18.2	16.3	23.3	37.7

Table S3: H3K27ac Metadata

Cell Type	Sample	Total Reads (millions)	Trimmed Reads (millions)	Aligned Reads (millions)	Alignment (%)	NRF	Peaks	FrIP (%)	NSC	RSC
BC	NC1	19.6	19.5	18.8	96.3	0.90	13,581	3.2	1.03	1.52
	NC2	16.4	15.4	14.2	92.2	0.62	19,319	4.2	1.11	4.23
	NC4	10.1	10.1	9.6	95.5	0.92	5,910	1.4	1.03	1.57
		17.7	17.4	16.7	95.7	0.91				
	NC Input	9.0	8.8	8.1	91.1	0.93	-	-	-	-
	MUT1	19.8	19.7	18.9	96.0	0.84	20,883	3.2	1.03	2.92
	MUT2	18.6	18.6	18.0	97.1	0.68	42,252	7.4	1.02	1.86
		16.2	16.1	15.7	97.1	0.70				
	MUT3	18.6	18.6	18.1	97.7	0.86	18,718	2.6	1.02	1.82
		13.6	13.6	13.2	97.2	0.87				
	MUT Input	16.7	16.7	15.3	91.6	0.88	-	-	-	-
LP	NC1	14.1	14.1	13.3	94.2	0.32	37,362	8.1	1.14	4.00
		16.9	16.8	15.9	94.6	0.29				
	NC2	22.8	22.3	21.2	95.2	0.86	53,218	23.3	1.18	1.41
		13.6	13.5	12.9	95.8	0.89				
	NC3	13.8	12.8	13.2	95.6	0.87	51,651	17.7	1.10	1.46
		22.1	21.9	21.0	96.0	0.87				
	NC4	5.2	5.2	5.0	96.4	0.94	49,647	21.9	1.15	1.48
		23.3	23.0	22.2	96.5	0.86				
		31.2	30.3	28.7	94.9	0.87				
	NC Input	15.3	15.2	14.0	92.0	0.88	-	-	-	-
	MUT1	14.6	14.6	14.0	96.3	0.86	52,641	15.7	1.07	1.55
		15.2	15.1	14.4	95.7	0.84				
		20.6	20.5	19.7	96.0	0.87				
	MUT2	13.0	13.0	12.7	97.2	0.70	70,990	21.1	1.08	1.63
		21.4	21.4	20.8	97.1	0.68				
	MUT3	14.8	14.7	14.3	97.0	0.87	40,357	9.2	1.05	1.82
		13.3	13.3	13.0	97.2	0.87				
	MUT Input	19.4	19.4	17.7	91.4	0.91	-	-	-	-
ML	NC1	12.1	11.7	11.0	93.8	0.34	21,702	5.8	1.14	3.83
		14.0	13.8	13.0	94.6	0.45				
	NC2	12.2	12.1	11.4	94.8	0.87	16,062	5.0	1.06	1.66
		16.2	16.0	15.2	95.1	0.84				
	NC3	19.6	19.5	18.9	96.8	0.89	31,709	12.2	1.08	1.45
		16.7	16.7	16.1	96.6	0.92				
	NC4	41.9	41.7	40.6	97.2	0.82	32,428	11.8	1.08	1.45
		44.3	43.7	42.4	97.0	0.86				

	NC Input	8.5	8.5	7.8	91.9	0.93	-	-	-	-
	MUT1	15.6	15.5	14.8	95.5	0.82	14,831	2.8	1.03	2.50
	MUT2	18.3	18.3	17.7	96.9	0.66	49,674	17.5	1.11	1.82
		17.9	17.9	17.4	97.1	0.67				
	MUT3	13.5	13.5	13.1	96.9	0.88	17,262	4.7	1.04	1.76
		14.0	14.0	13.6	97.1	0.84				
	MUT Input	11.5	11.5	10.5	91.4	0.90	-	-	-	-
SC	NC1	13.9	13.8	13.1	94.4	0.38	51,558	16.3	1.20	2.50
		13.6	13.5	12.9	95.6	0.40				
	NC2	17.3	17.2	16.6	96.7	0.87	44,403	15.8	1.13	1.58
		11.7	11.7	11.2	96.4	0.88				
	NC4	38.5	38.5	37.0	96.2	0.86	52,229	22.7	1.11	1.59
		28.4	28.4	27.5	96.8	0.90				
	NC Input	11.5	11.5	10.6	92.0	0.90	-	-	-	-
	MUT1	14.0	14.0	13.5	96.3	0.88	13,028	2.1	1.02	1.82
		13.1	13.0	12.4	95.8	0.88				
		13.0	13.0	12.2	93.7	0.87				
	MUT2	22.5	22.5	21.9	97.6	0.67	51,265	12.9	1.06	2.08
		16.7	16.7	16.3	97.4	0.72				
	MUT3	12.4	12.4	12.0	97.4	0.88	16,566	2.3	1.02	1.84
		13.4	13.3	13.0	97.5	0.87				
	MUT Input	16.5	16.5	15.1	91.8	0.91	-		-	-

Table S4: H3K4me3 Metadata

Cell Type	Sample	Total Reads (millions)	Trimmed Reads (millions)	Aligned Reads (millions)	Alignment (%)	NRF	Peaks	FrIP (%)	NSC	RSC
BC	NC5	17.3	17.0	16.1	94.8	0.81	27,186	16.1	1.14	1.66
		13.8	13.7	13.0	95.3	0.83				
	NC Input	9.0	8.8	8.1	91.1	0.93	-			
	MUT2	16.3	16.3	15.4	94.4	0.75	29,507	10.1	1.07	1.93
		15.8	15.8	14.9	94.5	0.76				
	MUT3	13.6	13.6	13.0	95.8	0.84	28,811	13.3	1.10	1.64
		14.7	14.7	14.0	95.5	0.84				
	MUT Input	16.7	16.7	15.3	91.6	0.88	-			
LP	NC5	18.4	18.4	17.4	94.6	0.74	33,050	16.0	1.14	1.80
	NC Input	15.3	15.2	14.0	92.0	0.88	-			
	MUT2	12.7	12.6	11.7	93.1	0.73	29,066	10.1	1.06	1.60
		13.3	13.3	12.4	93.4	0.79				
		12.9	12.9	12.0	93.3	0.77				
	MUT3	13.9	13.7	12.8	93.4	0.84	24,822	8.1	1.06	1.87
		12.6	12.5	11.9	94.9	0.85				
	MUT Input	19.4	19.4	17.7	91.4	0.91	-			
ML	NC5	13.1	12.9	12.3	96.9	0.82	22,748	11.9	1.12	1.67
		13.6	13.5	12.8	95.0	0.84				
	NC Input	8.5	8.5	7.8	91.9	0.93	-			
	MUT2	13.0	11.9	11.2	94.0	0.72	27,980	14.9	1.14	1.67
		15.4	14.2	13.1	92.8	0.73				
		13.8	12.8	12.0	93.6	0.74				
	MUT3	15.1	14.4	13.9	96.5	0.84	19,290	7.3	1.07	1.84
		12.6	12.0	11.6	96.6	0.85				
	MUT Input	11.5	11.5	10.5	91.4	0.90	-			
SC	NC5	11.3	11.3	10.7	94.8	0.82	25,504	15.3	1.16	1.82
		15.1	14.9	14.2	94.9	0.80				
	NC Input	11.5	11.5	10.6	92.0	0.90	-			
	MUT2	16.0	16.0	15.1	94.3	0.75	27,806	10.4	1.08	1.90
		13.8	13.8	13.0	94.2	0.79				
	MUT3	14.4	14.4	13.6	95.0	0.84	23,838	9.3	1.07	1.86
		12.3	12.3	11.7	95.3	0.87				
	MUT Input	16.5	16.5	15.1	91.8	0.91	-			

Table S5: Genes associated with differential regions in BCs between MUTs and NCs (in “Supplementary Tables_S4-S6.xlsx”)

Table S6: Genes associated with differential regions in LPs between MUTs and NCs (in “Supplementary Tables_S4-S6.xlsx”)

Table S7: Genes associated with differential regions in SCs between MUTs and NCs (in “Supplementary Tables_S4-S6.xlsx”)

Table S8: Significant Gene Ontology $-\log_{10}$(P-Values)

Gene Ontology	BC	SC
positive regulation of adherens junction organization	2.08	0
positive regulation of endothelial cell apoptotic process	1.98	0
positive regulation of focal adhesion assembly	2.26	0
negative regulation of adherens junction organization	2.47	0
cell-substrate junction assembly	2.37	0
negative regulation of focal adhesion assembly	2.36	0
regulation of endoplasmic reticulum unfolded protein response	4.82	0
regulation of vascular endothelial growth factor receptor signaling pathway	4.80	0
platelet-derived growth factor receptor signaling pathway	4.54	0
epithelial cell fate commitment	4.41	0
positive regulation of cyclic-nucleotide phosphodiesterase activity	4.38	0
lamellipodium assembly	3.74	0
positive regulation of vascular endothelial growth factor receptor signaling pathway	3.14	0
lamellipodium organization	3.30	0
positive regulation of cell junction assembly	3.45	0
progesterone metabolic process	7.83	3.37
regulation of cell shape	7.69	0
nuclear-transcribed mRNA catabolic process	3.46	9.88
regulation of transcription from RNA polymerase II promoter in response to stress	4.55	7.85
cellular response to unfolded protein	3.25	6.99
cellular response to topologically incorrect protein	3.24	8.27
endoplasmic reticulum unfolded protein response	3.74	7.90
interleukin-12-mediated signaling pathway	2.61	4.60
regulation of transcription from RNA polymerase II promoter in response to hypoxia	1.91	4.28
response to interleukin-12	2.31	4.18
"nuclear-transcribed mRNA catabolic process, deadenylation-dependent decay"	3.55	3.81
membrane protein proteolysis	3.02	2.26
positive regulation of chromosome segregation	0	1.86
TRIF-dependent toll-like receptor signaling pathway	0	2.05
response to arsenic-containing substance	0	2.21
vitamin transmembrane transport	0	2.16
DNA replication-independent nucleosome organization	0	3.07
ER-nucleus signaling pathway	0	3.07
DNA replication-independent nucleosome assembly	0	2.91
cellular response to arsenic-containing substance	0	2.81
histone H4 acetylation	0	2.85
"RNA phosphodiester bond hydrolysis, exonucleolytic"	0	2.42
membrane protein ectodomain proteolysis	0	2.30
cytochrome complex assembly	0	2.68
necroptotic process	0	2.58
negative regulation of protein tyrosine kinase activity	0	2.51

endosome to lysosome transport	0	3.37
positive regulation of protein insertion into mitochondrial membrane involved in apoptotic signaling pathway	0	3.65
response to laminar fluid shear stress	0	3.63
intrinsic apoptotic signaling pathway in response to DNA damage	0	3.70
regulation of mitochondrial outer membrane permeabilization involved in apoptotic signaling pathway	0	3.76
antigen processing and presentation of peptide antigen via MHC class I	0	4.20
positive regulation of mitochondrial outer membrane permeabilization involved in apoptotic signaling pathway	0	4.23
axo-dendritic transport	0	4.24
"antigen processing and presentation of exogenous peptide antigen via MHC class I, TAP-dependent"	0	4.25
antigen processing and presentation of exogenous peptide antigen via MHC class I	0	4.25
mRNA export from nucleus	0	4.94
regulation of mitochondrial membrane permeability	0	4.97
mitochondrial transmembrane transport	0	4.79
multi-organism transport	0	4.71

Table S9: Genomic Locations of Enhancers

Feature	BC (%)		LP (%)		ML (%)		SC (%)	
	NC	MUT	NC	MUT	NC	MUT	NC	MUT
<=1kb from promoter	17.9	7.6	8.8	7.2	12.7	8.3	10.3	11.6
1-2kb from promoter	6.8	4.8	4.8	4.6	5.2	4.6	5.5	5.4
5' UTR	0.8	1.1	1.0	1.1	1.0	1.1	1.1	0.8
3' UTR	2.4	2.9	2.7	2.9	2.8	2.7	3.2	2.8
1st Exon	0.9	0.6	0.6	0.6	0.8	0.6	0.6	0.7
Other Exon	3.1	4.7	4.6	5.0	4.3	4.7	4.5	4.1
1st Intron	16.7	16.5	17.1	15.8	17.3	17.8	16.9	15.9
Other Intron	19.3	23.1	22.7	23.1	21.5	24.3	25.3	23.5
Downstream (<=300)	1.4	1.2	1.2	1.2	1.5	1.4	1.2	1.1
Distal Intergenic	30.7	37.6	36.5	38.4	32.8	34.6	31.3	34.0

Table S10: Motif Enrichment -ln(P-Values)

Significant values (P< 0.0001) in green

Transcription Factor	BC		LP		ML		SC	
	NC	MUT	NC	MUT	NC	MUT	NC	MUT
AMYB	15.0	18.8	41.7	31.9	22.3	18.8	67.8	13.9
AP-1	162.0	120.0	1440.0	1330.0	599.0	822.0	878.0	276.0
AP-2alpha	12.6	47.1	97.4	174.0	63.4	73.9	44.5	24.7
AP-2gamma	10.2	70.8	104.0	174.0	64.2	68.7	56.0	29.0
Ap4	6.8	19.0	31.2	35.9	10.4	7.9	150.0	65.5
AR-halfsite	18.5	70.8	57.7	81.8	29.2	28.2	105.0	47.4
ARE	4.9	11.2	19.2	36.0	16.6	16.0	20.8	6.5
Arnt:Ahr	11.8	13.1	44.8	50.6	22.2	12.2	33.8	9.8
Ascl1	1.8	7.6	15.6	19.9	7.8	5.1	32.8	12.1
Atf1	36.0	29.4	115.0	134.0	86.6	114.0	131.0	28.4
Atf2	31.3	22.9	121.0	137.0	90.7	122.0	118.0	26.9
Atf3	177.0	125.0	1540.0	1440.0	671.0	941.0	953.0	292.0
Atf4	6.6	9.4	49.5	65.3	23.2	30.7	58.9	5.7
Atf7	35.0	29.1	125.0	138.0	97.2	140.0	152.0	33.3
Atoh1	5.4	17.4	18.3	16.3	1.2	1.1	108.0	53.5
Bach1	38.8	44.5	215.0	235.0	125.0	144.0	138.0	41.1
Bach2	103.0	64.9	765.0	745.0	338.0	486.0	404.0	115.0
Bapx1	12.5	15.8	24.2	17.5	10.7	9.7	54.8	16.2
Barx1	1.7	12.2	9.3	10.4	5.5	1.5	19.3	4.6
BATF	167.0	121.0	1520.0	1480.0	661.0	891.0	927.0	273.0
Bcl11a	6.9	34.2	33.8	33.9	11.8	18.7	41.2	30.6
Bcl6	25.2	79.9	188.0	216.0	52.6	44.5	189.0	71.4
bHLHE40	1.6	3.5	1.2	1.8	1.9	1.5	26.8	6.1
bHLHE41	5.3	6.0	9.9	18.8	17.9	23.8	13.0	2.1
BMAL1	7.4	33.5	35.6	38.3	13.1	16.3	168.0	41.5
BMYB	16.0	22.2	44.2	40.1	30.5	18.0	106.0	30.8
BORIS	0.2	4.9	6.2	4.2	2.6	6.1	16.4	7.0
Brachyury	4.9	13.2	10.9	17.7	2.1	3.5	17.9	10.1
Brn1	5.5	0.1	28.7	19.5	6.1	5.8	5.3	2.2
bZIP:IRF	20.0	21.4	88.4	69.3	38.4	42.5	93.7	17.9
c-Jun-CRE	33.1	27.0	148.0	160.0	104.0	131.0	126.0	29.5
c-Myc	2.6	6.8	0.4	4.3	0.8	2.5	36.8	15.5
c-Myc	0.3	16.0	7.6	3.0	0.3	2.5	53.5	19.7
CArG	14.2	27.7	18.5	34.0	11.5	5.9	41.4	19.2
Cdx2	4.0	6.4	20.2	52.6	15.2	5.2	27.8	9.3
CDX4	5.8	15.7	32.7	50.3	18.2	21.0	53.7	13.9
CEBP	14.2	21.4	139.0	204.0	56.4	40.0	212.0	31.5
CEBP:AP1	7.0	19.2	72.7	72.8	21.9	30.9	95.3	6.6
CEBP:CEBP	5.6	5.1	7.9	5.6	7.7	9.2	18.7	6.3

Chop	8.5	13.7	45.5	55.1	20.2	28.1	70.4	5.0
CHR	12.3	22.7	16.2	13.1	8.3	8.6	70.4	13.3
CLOCK	1.7	9.5	1.4	8.0	3.2	5.6	77.6	22.2
COUP-TFII	10.6	12.7	4.8	5.5	9.7	10.6	62.5	14.9
COUP-TFII	11.3	18.0	8.2	9.6	20.7	19.5	71.4	21.2
CRE	16.2	4.6	40.3	28.7	24.9	41.6	19.1	16.2
CRX	20.0	38.4	48.0	47.7	22.6	9.6	110.0	26.7
CTCF	1.8	8.5	4.8	13.3	3.2	6.5	24.2	10.4
Dlx3	7.7	24.5	39.9	29.5	16.9	8.8	18.5	15.5
DMRT1	2.1	2.7	5.5	21.7	3.2	1.7	7.5	3.6
DMRT6	2.4	2.0	3.0	12.1	3.3	2.8	1.8	1.8
E-box	5.1	3.4	2.8	2.7	5.7	3.8	23.9	3.6
E2A	0.0	0.3	8.2	19.7	6.2	9.6	0.0	0.0
E2F3	6.0	3.4	3.4	0.9	2.8	0.4	16.5	5.7
E2F6	2.0	6.5	5.5	3.3	2.5	1.4	17.0	8.8
EAR2	14.9	16.1	5.7	7.1	12.1	22.8	78.9	27.1
EBF	3.2	16.9	4.4	16.7	12.4	13.7	51.9	21.9
EBF1	14.2	42.6	47.7	70.6	39.4	27.3	112.0	44.7
EBF2	13.7	38.0	30.0	40.7	39.3	24.9	116.0	36.5
EBNA1	1.7	8.1	3.2	12.2	4.4	9.1	5.7	5.3
Egr1	2.5	15.8	20.1	6.9	3.4	2.1	38.4	31.9
Egr2	0.8	9.4	14.0	2.5	4.0	1.4	8.7	9.9
EHF	86.0	165.0	632.0	1010.0	220.0	240.0	134.0	107.0
EKLF	5.1	0.6	64.8	76.9	14.6	22.0	35.8	2.8
ELF1	30.0	82.9	277.0	417.0	119.0	145.0	59.3	110.0
ELF3	59.0	134.0	707.0	1090.0	260.0	278.0	135.0	101.0
Elf4	74.2	137.0	555.0	870.0	264.0	253.0	144.0	117.0
ELF5	37.9	99.5	592.0	861.0	212.0	221.0	98.0	96.7
Elk1	59.5	95.9	191.0	345.0	88.8	92.6	94.0	137.0
Elk4	67.2	101.0	158.0	253.0	74.3	72.1	78.1	110.0
Eomes	8.2	13.6	21.4	16.4	5.0	2.3	38.9	4.9
ERE	1.4	4.6	19.2	30.9	19.2	21.3	16.1	6.0
ERG	116.0	200.0	497.0	744.0	208.0	198.0	264.0	178.0
Erra	12.5	31.5	44.6	37.0	20.4	33.4	65.9	28.5
Esrrb	5.0	9.4	4.4	6.4	7.1	7.5	20.3	10.5
ETS	27.8	71.1	169.0	295.0	87.0	106.0	44.8	90.8
ETS:E-box	1.9	5.7	7.1	13.2	11.3	5.7	4.2	1.3
ETS:RUNX	8.0	23.4	24.0	61.6	7.9	14.4	6.0	9.5
Ets1-distal	107.0	170.0	204.0	320.0	105.0	90.3	210.0	166.0
ETS1	110.0	181.0	419.0	725.0	151.0	177.0	238.0	163.0
ETV1	104.0	201.0	482.0	759.0	201.0	214.0	206.0	162.0
Etv2	127.0	211.0	434.0	702.0	182.0	188.0	235.0	179.0
ETV4	115.0	197.0	369.0	610.0	174.0	170.0	215.0	174.0

EWS:ERG-fusion	150.0	199.0	432.0	632.0	146.0	156.0	260.0	173.0
EWS:FLI1-fusion	108.0	158.0	272.0	460.0	123.0	100.0	160.0	130.0
Fli1	144.0	183.0	375.0	670.0	173.0	175.0	239.0	179.0
Fosl2	163.0	109.0	1530.0	1500.0	681.0	939.0	836.0	253.0
Fox:Ebox	15.2	67.9	106.0	130.0	186.0	230.0	112.0	46.3
FOXA1	22.2	47.3	131.0	132.0	219.0	269.0	98.3	41.5
FOXA1	19.1	46.2	113.0	135.0	219.0	262.0	75.3	33.3
FOXA1:AR	2.3	3.9	7.7	14.7	13.6	18.4	3.1	2.5
Foxa2	22.5	44.6	117.0	116.0	216.0	251.0	73.8	43.2
Foxa3	19.5	31.8	80.3	87.3	145.0	167.0	39.1	24.3
Foxf1	32.9	44.0	119.0	162.0	142.0	154.0	104.0	64.6
Foxh1	9.2	21.0	32.6	32.3	10.3	13.5	52.6	28.1
FOXK1	44.5	52.9	115.0	156.0	154.0	175.0	141.0	82.2
FOXK2	27.3	57.3	115.0	113.0	126.0	146.0	140.0	57.3
FoxL2	26.1	35.7	104.0	120.0	141.0	165.0	109.0	58.9
FOXM1	13.1	48.4	105.0	98.4	209.0	247.0	77.1	36.7
Foxo1	51.5	93.8	206.0	261.0	130.0	163.0	230.0	101.0
Foxo3	42.6	48.6	117.0	155.0	160.0	188.0	133.0	71.4
FOXP1	31.5	40.4	110.0	175.0	131.0	179.0	113.0	58.8
Fra1	178.0	115.0	1590.0	1540.0	699.0	976.0	956.0	270.0
Fra2	183.0	115.0	1610.0	1540.0	705.0	990.0	897.0	260.0
FXR	5.4	9.6	8.3	0.3	4.1	4.3	12.5	9.4
GABPA	93.9	179.0	405.0	622.0	169.0	179.0	202.0	161.0
GATA	7.0	1.5	24.5	14.0	21.8	23.0	10.7	6.5
GATA	3.0	7.4	12.5	12.6	14.1	23.0	16.8	5.1
GATA:SCL	3.1	0.6	3.2	2.5	0.6	0.1	12.2	8.8
Gata1	9.3	5.9	0.1	0.0	2.1	6.7	33.8	10.8
Gata2	12.0	7.6	0.3	0.0	2.4	8.1	36.9	12.7
GATA3	1.9	2.3	11.3	5.5	8.1	3.7	2.8	1.6
GATA3	18.5	15.1	1.1	0.2	2.2	6.0	54.9	22.6
Gata4	16.9	15.2	0.6	0.0	2.1	7.8	61.9	17.3
Gata6	14.5	14.8	1.0	0.0	4.0	12.0	44.7	13.7
Gfi1b	9.3	22.1	32.4	34.5	12.1	5.0	51.8	16.0
GLIS3	5.7	14.9	15.8	15.3	12.5	16.0	68.9	24.3
GRE	7.0	12.2	21.3	25.3	14.5	10.9	26.5	7.5
GRE	10.7	7.9	17.1	14.4	22.8	16.3	35.1	10.4
GRHL2	1.8	16.7	187.0	214.0	97.0	101.0	13.2	3.8
GSC	10.9	34.5	44.6	52.4	15.3	13.6	111.0	25.3
Hand2	15.6	27.3	26.4	16.2	15.0	9.5	64.1	29.8
HEB	0.2	1.5	11.7	20.8	4.2	7.3	4.9	1.4
HIC1	20.2	116.0	154.0	183.0	41.1	38.7	65.4	14.1
HIF-1b	4.5	9.6	19.9	35.1	12.8	7.4	34.6	5.7
HIF2a	3.2	4.5	8.4	10.3	5.1	1.1	5.8	2.3

HLF	15.0	35.5	99.8	138.0	64.5	43.0	132.0	15.4
Hnf1	5.7	0.7	9.6	10.8	8.6	3.8	7.7	1.9
HNF1b	0.2	2.8	18.0	9.1	11.8	5.8	3.0	1.1
HNF4a	8.7	19.6	4.3	3.7	5.0	1.9	17.2	9.8
Hoxa10	11.7	15.5	32.7	51.5	7.3	5.6	20.5	14.8
Hoxa11	9.6	31.6	52.6	49.8	23.7	13.1	68.6	22.6
Hoxa13	11.5	27.2	48.2	65.1	26.4	16.4	85.1	36.5
Hoxa9	10.0	27.1	44.6	58.1	19.6	8.4	48.9	17.9
HOXB13	8.7	16.1	56.0	87.6	19.2	15.8	79.4	19.5
Hoxb4	7.3	6.1	26.5	11.8	7.7	11.0	7.3	6.4
Hoxc9	5.8	13.2	37.3	57.7	5.0	14.1	38.8	5.3
Hoxd10	17.5	28.3	41.0	60.2	14.2	8.9	49.9	29.0
Hoxd11	9.5	32.9	51.8	53.5	21.9	12.3	90.1	29.7
Hoxd12	11.5	21.9	51.0	41.2	12.0	5.6	63.9	26.0
Hoxd13	13.6	39.3	51.1	63.4	23.3	14.3	74.2	26.4
HSF1	6.8	12.4	10.9	7.0	1.9	1.8	14.2	6.9
HSF1	4.0	10.5	11.7	13.5	1.7	3.8	41.6	10.5
IRF:BATF	8.8	13.5	54.7	57.3	13.0	24.3	55.0	9.7
IRF1	44.7	40.0	94.2	105.0	62.9	55.1	185.0	56.1
IRF2	49.3	41.4	105.0	110.0	71.9	64.9	221.0	64.9
IRF3	63.6	49.3	136.0	152.0	67.3	45.2	194.0	58.5
IRF4	23.3	43.5	110.0	136.0	38.0	33.5	140.0	42.9
IRF8	48.8	36.6	130.0	145.0	79.7	57.2	181.0	59.3
Isl1	9.1	18.0	37.1	28.2	14.0	6.9	45.0	27.2
ISRE	43.7	29.7	81.6	83.3	63.1	54.8	152.0	52.4
Jun-AP1	154.0	107.0	1460.0	1370.0	666.0	869.0	718.0	226.0
JunB	173.0	117.0	1560.0	1490.0	695.0	927.0	906.0	282.0
JunD	13.1	11.9	82.9	73.6	69.5	70.8	49.0	17.9
KLF10	1.2	5.9	39.0	38.7	6.2	7.9	14.6	6.0
KLF14	13.8	8.6	36.2	38.2	10.2	13.2	77.9	19.5
KLF3	4.3	0.1	43.4	39.1	13.0	17.8	9.1	0.8
Klf4	5.9	0.8	60.7	76.6	14.8	24.5	21.2	0.9
KLF5	15.8	14.1	72.2	107.0	17.8	27.0	46.5	16.3
KLF6	17.4	5.2	61.4	91.0	19.6	28.8	46.8	14.0
Klf9	0.5	0.0	15.8	17.3	3.4	5.0	2.4	0.1
LEF1	18.5	52.1	36.0	39.3	12.5	12.2	35.5	15.9
Lhx1	10.3	36.3	39.4	38.2	13.3	6.4	40.0	27.7
Lhx2	6.9	27.7	54.2	33.5	18.3	6.9	37.7	21.5
Lhx3	8.7	30.0	52.3	54.5	15.4	10.6	58.4	32.0
LRF	2.4	9.5	0.0	0.0	0.0	0.0	16.2	8.4
LXRE	4.8	3.8	3.2	1.4	3.8	0.9	10.3	5.6
MafA	23.1	36.6	63.6	87.7	31.4	54.9	115.0	34.4
MafB	34.3	22.7	86.3	90.4	37.7	41.9	121.0	31.9

MafF	5.5	12.7	10.0	16.1	9.9	4.2	49.9	19.4
MafK	34.7	22.7	177.0	177.0	81.7	115.0	183.0	49.5
Max	1.5	21.9	3.1	9.0	1.4	6.6	110.0	29.6
Maz	21.1	4.5	25.1	8.6	15.8	12.5	63.3	18.6
Mef2a	19.3	33.9	27.1	39.2	7.8	8.9	89.5	25.8
Mef2b	31.7	52.2	47.2	53.2	14.8	11.9	106.0	48.0
Mef2c	28.7	28.7	37.8	37.0	11.8	14.2	127.0	42.1
Mef2d	22.4	34.9	30.2	29.8	10.7	10.9	118.0	44.5
Meis1	1.1	7.3	30.7	24.0	7.0	6.9	45.6	7.3
MITF	20.1	47.1	39.7	59.1	30.6	27.4	201.0	42.6
MNT	1.8	18.6	5.2	7.3	1.2	3.2	102.0	32.1
MYB	20.4	16.0	36.1	33.1	18.1	13.2	87.9	24.2
Myf5	3.6	4.7	13.1	27.5	4.0	3.8	65.1	22.6
MYNN	7.6	7.4	19.2	19.9	4.8	5.7	34.5	4.9
MyoD	4.4	3.8	18.7	23.7	6.9	5.5	75.5	23.5
MyoG	1.6	6.9	21.3	37.5	8.0	8.7	55.0	23.1
n-Myc	0.4	17.4	8.9	6.8	1.5	4.1	82.6	25.9
Nanog	5.4	15.7	46.1	29.5	13.2	2.8	53.9	16.5
NeuroD1	8.6	23.2	6.6	10.1	4.5	3.2	89.1	39.4
NeuroG2	4.2	19.3	20.0	14.5	1.8	2.8	132.0	66.9
NF-E2	63.8	49.4	242.0	285.0	173.0	205.0	157.0	64.9
NF1-halfsite	31.0	233.0	400.0	507.0	102.0	90.8	213.0	52.6
NF1	30.9	151.0	286.0	369.0	72.6	87.3	224.0	62.0
NF1:FOXA1	7.3	13.7	70.3	115.0	21.1	20.9	29.9	14.4
NFAT	9.3	25.9	59.5	55.5	16.8	14.9	175.0	62.0
NFAT:AP1	6.7	13.7	49.1	68.3	8.3	9.1	92.9	45.2
NFE2L2	44.9	45.8	191.0	185.0	115.0	122.0	125.0	60.6
NFIL3	24.2	36.4	162.0	177.0	77.2	82.4	156.0	23.1
NFkB-p50,p52	11.8	13.1	7.1	20.3	9.7	8.4	15.4	5.4
NFkB-p65-Rel	38.9	39.4	152.0	138.0	43.8	47.7	47.5	34.5
NFkB-p65	61.3	38.7	149.0	198.0	55.1	68.5	139.0	64.6
Nkx2.1	10.6	27.3	25.2	27.9	8.4	7.4	71.9	18.1
Nkx2.2	12.2	15.0	4.3	2.7	7.2	9.5	45.1	15.5
Nkx2.5	16.0	29.5	9.2	14.9	6.4	5.9	68.7	15.5
Nkx3.1	8.7	46.2	35.4	55.9	16.7	19.4	72.2	19.1
Nkx6.1	6.5	29.1	63.9	62.2	31.9	16.4	83.1	28.8
NPAS	5.4	28.9	16.8	28.8	4.3	8.1	126.0	31.6
NPAS2	1.1	11.0	7.0	20.3	4.8	12.7	55.1	13.3
Nr5a2	5.0	24.8	8.1	16.7	2.2	6.3	24.7	24.7
Nr5a2	6.1	22.2	6.1	13.5	1.9	2.5	18.9	17.5
Nrf2	48.2	36.7	195.0	225.0	135.0	166.0	125.0	42.9
Nur77	5.4	2.2	6.9	10.0	10.8	9.5	10.4	3.7
OCT:OCT-short	1.4	1.4	10.6	9.6	2.5	0.8	5.1	0.2

Oct2	1.3	0.2	10.5	2.3	6.1	7.3	3.1	0.4
Oct4	1.4	0.0	18.9	3.9	6.1	5.2	3.3	1.4
Oct6	4.6	0.3	26.1	23.6	5.6	5.4	19.3	5.4
Olig2	6.1	23.3	17.7	6.3	0.9	1.1	111.0	55.0
Otx2	11.0	33.3	28.5	45.1	14.0	9.4	69.4	12.6
p53	3.2	26.0	0.4	7.8	2.5	0.9	1.7	1.6
p53	10.9	214.0	10.1	14.7	11.7	6.6	7.8	2.9
p63	10.1	216.0	22.8	32.8	11.1	11.1	22.6	3.5
p73	7.4	191.0	8.4	7.1	13.9	14.4	18.6	3.3
PAX3:FKHR-fusion	3.2	2.9	24.8	17.5	5.5	8.5	20.4	5.5
PAX5	3.9	5.8	5.9	7.3	2.1	4.4	10.7	1.9
PAX5	2.1	11.8	14.3	15.4	16.9	8.7	20.5	7.0
Pax8	5.2	18.1	18.2	26.8	4.0	10.7	26.8	10.2
PBX2	3.6	9.2	34.1	32.3	11.1	15.6	23.1	7.7
Pdx1	3.8	14.3	61.0	58.2	14.3	16.7	42.0	9.7
PGR	12.8	16.2	30.6	19.2	28.0	19.1	58.1	19.6
Phox2a	7.3	7.9	6.7	5.5	2.7	1.4	16.9	7.2
Pit1	0.3	5.1	12.5	7.9	0.7	0.7	2.1	0.1
Pitx1	30.0	52.6	91.1	88.7	39.9	21.5	123.0	50.5
Pitx1:Ebox	1.7	9.7	15.0	15.5	0.9	1.7	223.0	104.0
PPARa	18.8	19.2	26.7	18.6	23.7	16.0	29.1	18.7
PPARE	18.2	30.8	29.7	33.6	21.7	20.0	40.1	41.4
PR	13.6	50.8	80.4	61.5	24.1	8.8	114.0	49.3
PRDM1	36.3	49.5	67.7	41.0	49.7	34.0	118.0	38.4
PRDM10	3.3	23.4	14.8	22.4	6.5	8.0	9.0	5.9
PRDM14	4.8	5.6	5.0	16.3	0.6	1.3	12.5	2.6
PRDM15	12.4	29.0	29.1	44.9	11.2	17.5	67.1	19.2
PRDM9	0.4	0.2	0.2	2.1	0.2	0.1	9.7	1.9
Prop1	6.7	9.6	11.5	9.2	3.1	1.8	21.1	9.2
PSE	7.4	5.7	21.6	29.4	9.4	14.6	25.7	12.5
Ptf1a	1.0	3.4	11.6	13.2	6.9	5.4	25.6	9.2
PU.1-IRF	50.5	67.9	123.0	118.0	43.3	45.4	154.0	77.3
PU.1	73.2	83.7	206.0	313.0	77.3	89.3	123.0	63.1
PU.1:IRF8	33.1	25.2	78.4	91.5	47.2	29.5	117.0	37.6
RARa	12.1	26.5	27.8	35.8	30.3	25.5	106.0	42.5
RBPJ:Ebox	5.7	21.0	25.7	28.2	5.0	17.3	31.4	16.6
Rbpj1	26.9	46.1	121.0	97.8	36.7	47.8	170.0	70.0
Reverb	4.4	7.9	11.2	3.7	6.0	6.4	15.1	13.6
RFX	17.6	55.4	35.9	74.5	49.5	51.3	26.3	27.8
Rfx1	18.6	52.2	41.2	70.6	32.3	35.5	28.7	29.1
Rfx2	15.0	63.3	34.0	70.5	42.2	48.3	20.4	23.0
Rfx5	27.7	64.3	61.9	97.5	47.3	49.6	41.3	28.8
Rfx6	22.4	89.2	117.0	174.0	42.7	35.7	146.0	45.8

RORa	11.3	41.1	17.8	44.3	11.1	15.2	54.7	38.2
RORg	9.7	19.4	12.2	28.0	9.7	18.1	31.6	25.6
RORgt	6.3	25.0	15.7	24.5	15.0	16.9	52.6	28.5
RUNX-AML	12.7	48.0	97.9	77.6	19.8	15.7	82.9	49.6
RUNX	14.8	49.3	119.0	82.2	26.7	28.7	81.2	46.2
RUNX1	10.0	61.0	132.0	121.0	33.0	30.0	111.0	53.4
RUNX2	14.0	52.3	96.4	70.3	16.2	18.3	92.6	53.2
RXR	11.9	28.1	23.0	35.7	25.3	18.4	30.3	27.0
SCL	11.4	27.2	21.3	28.5	10.0	7.5	89.5	17.0
SCRT1	0.2	1.2	7.5	1.2	6.8	13.0	0.0	0.0
SF1	3.3	14.9	5.4	15.6	1.6	3.9	17.3	8.7
Six1	7.1	11.3	15.3	33.1	21.5	8.1	46.2	10.4
Six2	18.6	40.7	44.6	55.9	28.1	16.1	68.7	13.0
Six4	1.0	1.8	10.0	9.5	7.8	1.0	11.8	5.8
Smad2	18.6	55.9	68.6	72.3	23.3	11.2	143.0	46.3
Smad3	30.4	82.7	96.0	116.0	25.9	17.8	188.0	58.0
Smad4	11.1	51.6	63.5	48.2	20.7	13.9	127.0	27.9
Sox10	35.2	85.9	248.0	310.0	69.3	60.2	50.1	27.8
Sox15	34.6	109.0	283.0	291.0	59.1	51.5	44.6	37.7
Sox17	24.2	65.0	178.0	172.0	29.7	22.0	16.8	14.1
Sox2	41.4	131.0	273.0	313.0	55.0	49.6	55.0	41.3
Sox3	36.2	99.4	269.0	297.0	68.4	53.5	53.5	28.3
Sox4	31.8	68.1	228.0	284.0	79.8	71.5	50.3	24.0
Sox6	33.9	95.2	240.0	277.0	54.0	52.8	64.0	31.2
Sox9	20.2	71.3	156.0	223.0	36.7	36.2	55.0	27.9
Sp1	0.9	0.0	4.8	10.1	6.1	5.3	0.0	0.0
Sp2	19.7	12.0	36.8	31.7	19.0	10.6	39.2	14.8
Sp5	15.7	2.3	34.0	33.2	13.7	15.0	51.6	11.6
SPDEF	69.5	117.0	371.0	575.0	121.0	129.0	125.0	67.5
SpiB	20.3	31.3	90.8	91.9	28.0	35.4	47.0	42.4
Srebp1a	4.2	4.5	5.9	4.8	5.5	5.4	18.3	6.5
Srebp2	6.6	6.4	2.6	2.7	4.0	5.5	16.7	11.6
STAT1	15.6	37.2	91.2	124.0	35.6	67.1	107.0	28.6
Stat3	35.0	85.6	221.0	256.0	99.1	122.0	134.0	35.5
Stat3+il21	41.4	95.2	192.0	247.0	102.0	110.0	163.0	56.3
STAT4	37.0	83.6	152.0	240.0	76.9	85.2	181.0	69.6
STAT5	17.6	33.6	83.7	101.0	26.2	54.7	89.5	29.1
STAT6	11.0	28.2	29.0	39.3	7.7	7.4	67.1	25.7
STAT6	11.0	29.1	31.0	38.2	7.8	8.0	76.5	26.5
T1ISRE	5.4	1.8	10.5	6.9	1.5	2.3	8.7	1.3
TATA-Box	19.7	50.9	53.2	80.8	21.9	19.5	134.0	44.7
Tbet	3.5	8.7	10.8	23.3	1.4	2.2	5.9	5.3
Tbox:Smad	4.2	17.5	9.0	6.7	4.3	4.7	8.3	1.8

Tbr1	5.1	9.8	39.8	33.1	5.2	4.1	26.3	8.7
Tbx20	1.0	5.6	9.8	12.0	7.2	8.2	9.0	1.2
Tbx21	6.0	9.7	17.0	21.2	3.2	1.1	6.4	4.3
Tbx5	3.4	21.8	44.6	60.0	12.6	10.0	13.9	1.1
Tbx6	2.4	3.8	15.2	9.4	1.4	1.0	0.2	0.0
Tcf12	3.8	7.1	14.0	20.9	5.7	8.4	62.6	16.5
Tcf21	8.2	16.7	24.5	26.3	5.6	3.5	168.0	62.4
Tcf3	7.9	25.9	5.1	6.9	5.3	3.6	4.2	4.3
TCF4	6.9	21.4	6.1	8.3	2.5	2.2	135.0	72.3
Tcf7	5.4	30.1	6.3	6.5	8.3	6.4	8.5	6.8
Tcfcp2l1	14.6	53.5	21.3	30.1	18.4	15.7	28.8	9.8
TCFL2	1.4	11.5	3.5	4.5	4.6	2.6	6.2	2.0
TEAD	32.4	134.0	262.0	178.0	44.6	50.5	103.0	29.9
TEAD1	44.8	152.0	264.0	186.0	50.4	54.5	109.0	34.1
TEAD2	44.7	127.0	210.0	147.0	33.9	39.3	69.1	18.7
TEAD3	37.3	144.0	229.0	167.0	49.7	48.9	98.3	35.1
TEAD4	47.4	142.0	282.0	177.0	60.2	57.2	99.3	33.6
TFE3	2.7	3.2	3.3	2.9	6.4	5.3	13.8	7.7
Tgif1	5.3	18.1	41.6	40.1	26.8	13.5	79.6	23.1
Tgif2	8.1	25.6	57.4	69.2	28.7	20.3	78.9	20.1
THRa	9.4	15.1	4.3	5.9	1.3	3.8	29.0	25.8
THRb	7.9	11.2	3.7	6.7	3.7	6.4	42.4	26.0
THRb	20.3	57.2	34.6	45.0	21.1	23.7	85.1	36.2
Tlx?	17.9	118.0	211.0	260.0	53.8	65.2	185.0	45.6
Twist	0.4	1.2	0.1	0.6	0.6	0.2	16.7	8.8
Unknown-ESC-element	4.8	13.9	9.4	15.0	3.9	7.9	21.3	7.5
Unknown	12.2	25.1	28.7	35.6	20.2	13.0	38.6	22.7
USF1	2.9	10.5	3.7	17.6	5.4	9.4	109.0	30.1
Usf2	4.0	13.8	6.5	28.3	12.4	15.6	84.9	16.4
WT1	5.2	24.5	15.5	10.4	13.9	7.8	44.2	28.1
X-box	20.5	43.9	31.1	58.7	28.1	34.9	33.8	30.4
Zac1	7.1	33.2	12.2	7.9	2.9	3.6	75.0	30.6
ZBTB12	0.8	1.8	5.2	2.3	0.5	0.8	22.2	6.0
ZBTB18	4.0	3.6	3.2	3.6	0.1	0.2	89.5	40.5
Zfp281	4.1	0.0	15.1	10.6	9.6	7.8	18.5	3.2
Zfp809	3.1	17.5	12.0	20.2	11.9	8.8	18.0	9.8
ZFX	10.8	101.0	46.8	60.7	16.9	27.4	123.0	60.6
Zic	0.6	6.5	3.8	8.4	0.5	8.2	11.6	2.5
Zic3	1.7	3.2	2.7	9.7	1.9	3.9	11.0	6.7
ZNF136	0.7	0.4	7.4	6.1	2.2	0.2	11.7	1.7
ZNF143|STAF	4.0	21.8	5.3	6.4	1.3	2.3	33.8	12.7
ZNF189	11.3	18.4	31.9	37.8	11.6	11.0	62.9	31.0
Znf263	3.9	26.9	13.5	10.6	6.8	4.7	37.4	15.7

ZNF264	2.8	11.2	9.1	8.8	4.2	1.9	5.0	1.2
ZNF317	2.9	3.3	7.4	6.3	7.5	6.6	24.7	5.6
ZNF322	2.6	19.0	16.7	21.3	2.6	7.0	31.5	6.1
ZNF382	1.8	2.5	5.9	12.3	0.7	0.7	4.7	1.1
ZNF415	0.6	3.7	1.4	0.6	0.6	0.1	11.3	3.8
ZNF416	22.9	78.1	89.9	131.0	48.6	46.2	86.8	33.1
ZNF467	14.0	16.5	16.7	17.9	17.5	8.2	48.6	16.8
ZNF528	3.1	2.0	4.2	10.4	1.8	1.7	3.9	1.4
ZNF652	7.8	16.2	23.2	23.5	1.7	4.5	24.2	11.0
ZNF675	4.0	12.0	7.2	10.7	3.1	1.6	17.1	5.4
ZNF7	4.3	15.2	20.1	16.9	5.0	8.1	47.8	11.3
ZNF711	16.8	86.3	43.5	62.3	26.3	26.6	134.0	61.9
ZSCAN22	2.0	3.4	3.7	10.3	2.5	1.7	14.1	12.7

Table S11: Number of differential transcription factors annotated to Panther pathways

	BC	LP	ML	SC
Alzheimer disease-presenilin pathway	2	0	1	0
Angiogenesis	3	0	1	1
Angiotensin II-stimulated signaling through G proteins and beta-arrestin	1	1	0	0
Apoptosis signaling pathway	3	2	3	3
B cell activation	2	2	2	2
Blood coagulation	1	0	0	0
CCKR signaling map	4	4	2	5
Cell cycle	0	0	0	1
Circadian clock system	2	0	0	0
EGF receptor signaling pathway	1	0	0	0
FAS signaling pathway	1	0	0	1
Gonadotropic-releasing hormone receptor pathway	10	5	4	3
Huntington disease	3	1	1	3
Hypoxia respose via HIF activation	2	0	0	0
Inflammation mediated by chemokine and cytokine signaling pathway	2	2	2	2
Insulin/IGF pathway-protein kinase B signaling cascade	0	0	0	1
Interleuking signaling pathway	1	1	2	1
Notch signaling pathway	1	0	1	0
Oxidative stress response	4	1	1	3
p53 pathway feedback loops 1	2	0	1	2
PDGF signaling pathway	4	3	3	3
PI3 kinase pathway	0	0	0	1
Ras pathway	1	0	1	1
T cell activation	2	2	2	2
TGF-beta signaling pathway	2	0	0	1
Toll receptor signaling pathway	1	2	2	2
Wnt signaling pathway	6	1	2	1
p38 MAPK pathway	0	0	1	0
p53 pathway by glucose deprivation	2	0	1	2
p53 pathway feedback loops 2	3	1	2	4
p53 pathway	2	0	1	3

Table S12: Cell-Type-Specific Genes Significantly Marked Between NC and MUT BCs

Ensembl ID	Gene Name	Fold Change (NC/MUT)	FDR	Cell-Type	Directionality
ENSG00000043462	LCP2	4.72	1.35E-05	BC	UP
ENSG00000059804	SLC2A3	2.09	2.15E-03	BC	UP
ENSG00000091831	ESR1	3.42	2.96E-02	BC	DOWN
ENSG00000091879	ANGPT2	3.17	5.78E-08	BC	UP
ENSG00000118407	FILIP1	2.57	3.91E-04	BC	UP
ENSG00000119865	CNRIP1	2.13	3.91E-04	BC	UP
ENSG00000120279	MYCT1	3.86	3.09E-02	BC	UP
ENSG00000121797	CCRL2	2.66	3.27E-05	BC	UP
ENSG00000125810	CD93	2.5	9.93E-06	BC	UP
ENSG00000125845	BMP2	2.11	1.23E-02	BC	UP
ENSG00000125878	TCF15	3.11	2.27E-02	BC	UP
ENSG00000128652	HOXD3	2.79	3.06E-05	BC	UP
ENSG00000130300	PLVAP	3.01	3.27E-05	BC	UP
ENSG00000132622	HSPA12B	2.03	2.87E-02	BC	UP
ENSG00000139567	ACVRL1	2.07	5.06E-04	BC	UP
ENSG00000144837	PLA1A	2.17	5.56E-04	BC	UP
ENSG00000147113	CXorf36	3.5	3.73E-06	BC	UP
ENSG00000149564	ESAM	2.6	3.91E-04	BC	UP
ENSG00000155962	CLIC2	3.28	8.95E-06	BC	UP
ENSG00000157510	AFAP1L1	2.09	1.31E-03	BC	UP
ENSG00000160349	LCN1	3.87	8.19E-03	BC	UP
ENSG00000161940	BCL6B	2.37	8.95E-06	BC	UP
ENSG00000162444	RBP7	2.29	3.39E-02	BC	UP
ENSG00000162654	GBP4	2.14	2.07E-03	BC	DOWN
ENSG00000170323	FABP4	2.85	1.34E-03	BC	UP
ENSG00000170989	S1PR1	2.71	2.87E-02	BC	UP
ENSG00000171115	GIMAP8	2.28	6.79E-05	BC	UP
ENSG00000172889	EGFL7	2.7	2.90E-02	BC	UP
ENSG00000174175	SELP	4.88	3.43E-03	BC	UP
ENSG00000175879	HOXD8	3.27	8.95E-06	BC	UP
ENSG00000176435	CLEC14A	2.82	6.45E-05	BC	UP
ENSG00000177464	GPR4	2.09	1.30E-02	BC	UP
ENSG00000178175	ZNF366	2.75	3.27E-05	BC	UP
ENSG00000179144	GIMAP7	2.92	1.72E-02	BC	UP
ENSG00000196329	GIMAP5	3.01	2.39E-04	BC	UP
ENSG00000198844	ARHGEF15	2.43	5.56E-04	BC	UP
ENSG00000203883	SOX18	2.29	2.07E-03	BC	UP
ENSG00000204291	COL15A1	2.39	5.38E-03	BC	UP
ENSG00000213088	DARC	2.48	2.21E-03	BC	UP
ENSG00000242550	SERPINB10	2.37	4.31E-02	BC	UP

ENSG00000249751	ECSCR	2.51	1.55E-05	BC	UP
ENSG00000021852	C8B	3.19	3.24E-02	ML	UP
ENSG00000105388	CEACAM5	3.47	2.87E-02	ML	UP
ENSG00000105971	CAV2	2.87	2.96E-02	ML	DOWN
ENSG00000144218	AFF3	4.06	1.20E-02	ML	UP
ENSG00000150630	VEGFC	2.13	5.74E-03	ML	UP
ENSG00000153071	DAB2	3.97	5.79E-04	ML	DOWN
ENSG00000162692	VCAM1	2.06	1.00E-02	ML	DOWN
ENSG00000163534	FCRL1	3.21	4.85E-02	ML	UP
ENSG00000171428	NAT1	4.02	1.72E-02	ML	UP
ENSG00000180777	ANKRD30B	4.11	1.80E-02	ML	UP
ENSG00000227921	AL353791.1	4.19	1.38E-02	ML	DOWN
ENSG00000137441	FGFBP2	3.31	3.24E-02	LP	UP
ENSG00000171209	CSN3	3.92	3.88E-02	LP	UP
ENSG00000196743	GM2A	4.03	1.88E-02	LP	DOWN
ENSG00000205502	C2CD4B	3.19	4.88E-02	LP	DOWN

Table S13: Cell-Type-Specific Motif Enrichment -ln(P-Values)

Significant values (P<0.0001) in green

Transcription Factor	NC			MUT		
	BC	LP	ML	BC	LP	ML
Ascl1	1.8	15.6	7.8	7.6	19.9	5.1
Atoh1	5.4	18.3	1.2	17.4	16.3	1.1
Barx1	1.7	9.3	5.5	12.2	10.4	1.5
Brachyury	4.9	10.9	2.1	13.2	17.7	3.5
Brn1	5.5	28.7	6.1	0.1	19.5	5.8
EBF	3.2	4.4	12.4	16.9	16.7	13.7
Egr1	2.5	20.1	3.4	15.8	6.9	2.1
Egr2	0.8	14.0	4.0	9.4	2.5	1.4
Eomes	8.2	21.4	5.0	13.6	16.4	2.3
ETS:E-box	1.9	7.1	11.3	5.7	13.2	5.7
ETS:RUNX	8.0	24.0	7.9	23.4	61.6	14.4
FOXA1:AR	2.3	7.7	13.6	3.9	14.7	18.4
Gata1	9.3	0.1	2.1	5.9	0.0	6.7
Gata2	12.0	0.3	2.4	7.6	0.0	8.1
GATA3	1.9	11.3	8.1	2.3	5.5	3.7
GATA3	18.5	1.1	2.2	15.1	0.2	6.0
Gata4	16.9	0.6	2.1	15.2	0.0	7.8
Gata6	14.5	1.0	4.0	14.8	0.0	12.0
HEB	0.2	11.7	4.2	1.5	20.8	7.3
Hnf1	5.7	9.6	8.6	0.7	10.8	3.8
Hoxb4	7.3	26.5	7.7	6.1	11.8	11.0
Hoxc9	5.8	37.3	5.0	13.2	57.7	14.1
HSF1	6.8	10.9	1.9	12.4	7.0	1.8
HSF1	4.0	11.7	1.7	10.5	13.5	3.8
KLF10	1.2	39.0	6.2	5.9	38.7	7.9
Klf9	0.5	15.8	3.4	0.0	17.3	5.0
Meis1	1.1	30.7	7.0	7.3	24.0	6.9
Myf5	3.6	13.1	4.0	4.7	27.5	3.8
MYNN	7.6	19.2	4.8	7.4	19.9	5.7
MyoD	4.4	18.7	6.9	3.8	23.7	5.5
MyoG	1.6	21.3	8.0	6.9	37.5	8.7
NeuroG2	4.2	20.0	1.8	19.3	14.5	2.8
NFAT:AP1	6.7	49.1	8.3	13.7	68.3	9.1
Nkx2.2	12.2	4.3	7.2	15.0	2.7	9.5
Nkx2.5	16.0	9.2	6.4	29.5	14.9	5.9
NPAS	5.4	16.8	4.3	28.9	28.8	8.1
Nur77	5.4	6.9	10.8	2.2	10.0	9.5
OCT:OCT-short	1.4	10.6	2.5	1.4	9.6	0.8
Oct2	1.3	10.5	6.1	0.2	2.3	7.3

Oct4	1.4	18.9	6.1	0.0	3.9	5.2
Oct6	4.6	26.1	5.6	0.3	23.6	5.4
Olig2	6.1	17.7	0.9	23.3	6.3	1.1
p73	7.4	8.4	13.9	191.0	7.1	14.4
PAX3:FKHR-fusion	3.2	24.8	5.5	2.9	17.5	8.5
Pax8	5.2	18.2	4.0	18.1	26.8	10.7
Pit1	0.3	12.5	0.7	5.1	7.9	0.7
Pitx1:Ebox	1.7	15.0	0.9	9.7	15.5	1.7
PRDM10	3.3	14.8	6.5	23.4	22.4	8.0
Prop1	6.7	11.5	3.1	9.6	9.2	1.8
Ptf1a	1.0	11.6	6.9	3.4	13.2	5.4
RBPJ:Ebox	5.7	25.7	5.0	21.0	28.2	17.3
Reverb	4.4	11.2	6.0	7.9	3.7	6.4
Six4	1.0	10.0	7.8	1.8	9.5	1.0
T1ISRE	5.4	10.5	1.5	1.8	6.9	2.3
Tbet	3.5	10.8	1.4	8.7	23.3	2.2
Tbr1	5.1	39.8	5.2	9.8	33.1	4.1
Tbx20	1.0	9.8	7.2	5.6	12.0	8.2
Tbx21	6.0	17.0	3.2	9.7	21.2	1.1
Tbx6	2.4	15.2	1.4	3.8	9.4	1.0
Tcf12	3.8	14.0	5.7	7.1	20.9	8.4
Tcf21	8.2	24.5	5.6	16.7	26.3	3.5
THRa	9.4	4.3	1.3	15.1	5.9	3.8
Unknown-ESC-element	4.8	9.4	3.9	13.9	15.0	7.9
Usf2	4.0	6.5	12.4	13.8	28.3	15.6
Zac1	7.1	12.2	2.9	33.2	7.9	3.6
Znf263	3.9	13.5	6.8	26.9	10.6	4.7
ZNF322	2.6	16.7	2.6	19.0	21.3	7.0
ZNF652	7.8	23.2	1.7	16.2	23.5	4.5
ZNF7	4.3	20.1	5.0	15.2	16.9	8.1
